# Post-duplication charge evolution of phosphoglucose isomerases in teleost fishes through weak selection on many amino acid sites

**DOI:** 10.1186/1471-2148-7-204

**Published:** 2007-10-29

**Authors:** Yukuto Sato, Mutsumi Nishida

**Affiliations:** 1 Division of Molecular Marine Biology, Ocean Research Institute, The University of Tokyo, 1-15-1 Minamidai, Nakano-ku, Tokyo 164-8639, Japan; 2 Department of Aquatic Bioscience, Graduate School of Agricultural and Life Sciences, The University of Tokyo, 1-1-1 Yayoi, Bunkyo-ku, Tokyo 113-8657, Japan

## Abstract

**Background:**

The partitioning of ancestral functions among duplicated genes by neutral evolution, or subfunctionalization, has been considered the primary process for the evolution of novel proteins (neofunctionalization). Nonetheless, how a subfunctionalized protein can evolve into a more adaptive protein is poorly understood, mainly due to the limitations of current analytical methods, which can detect only strong selection for amino acid substitutions involved in adaptive molecular evolution. In this study, we employed a comparative evolutionary approach to this question, focusing on differences in the structural properties of a protein, specifically the electric charge, encoded by fish-specific duplicated phosphoglucose isomerase (*Pgi*) genes.

**Results:**

Full-length cDNA cloning, RT-PCR based gene expression analyses, and comparative sequence analyses showed that after subfunctionalization with respect to the expression organ of duplicate *Pgi *genes, the net electric charge of the PGI-1 protein expressed mainly in internal tissues became more negative, and that of PGI-2 expressed mainly in muscular tissues became more positive. The difference in net protein charge was attributable not to specific amino acid sites but to the sum of various amino acid sites located on the surface of the PGI molecule.

**Conclusion:**

This finding suggests that the surface charge evolution of PGI proteins was not driven by strong selection on individual amino acid sites leading to permanent fixation of a particular residue, but rather was driven by weak selection on a large number of amino acid sites and consequently by steady directional and/or purifying selection on the overall structural properties of the protein, which is derived from many modifiable sites. The mode of molecular evolution presented here may be relevant to various cases of adaptive modification in proteins, such as hydrophobic properties, molecular size, and electric charge.

## Background

Proteins that arise through gene duplication can become novel proteins through fixation of beneficial mutations [1], but because beneficial mutations are generally rare, the partitioning of ancestral functions among duplicated genes by neutral evolution, or subfunctionalization, has been considered the primary process for the evolution of novel proteins [2-5]. To date, many duplicate genes have been demonstrated to evolve following this model of subfunctionalization, and this model thus has become widely accepted in the context of duplicated gene evolution [6-10].

Nonetheless, how a more adaptive or specialized protein property evolves after subfunctionalization is poorly understood, mainly due to the limited resolution power of current analytical methods, which seek to detect positive selection on individual amino acid substitutions involved in adaptive molecular evolution. Such methods can recognize substitutions expected to be driven by strong selection, many of which are usually function-altering substitutions at important amino acid sites, such as enzyme active sites or viral epitopes [e.g., [11]]. However, adaptive substitutions by relatively moderate or weak selection may not be recognized by these methods. Therefore, to detect adaptive protein evolution under a much wider range of selection pressure, a novel approach is required. In this study, we utilized a comparative evolutionary approach to this problem, focusing on differences in the high-dimensional properties of a protein, specifically the electric charge, encoded by a pair of duplicated genes.

As a model protein system for this study, we chose an important enzyme involved in glycolysis and gluconeogenesis, phosphoglucose isomerase (PGI; EC 5.3.1.9). The gene encoding this enzyme (*Pgi*) is present as a single copy in tetrapods, whereas two copies exist in most groups of ray-finned fishes [12-14]. The fact that these duplicated *Pgi-1 *and *Pgi-2 *genes in fishes are expressed in different organs [12,15] implies that these fish-specific duplicate *Pgi *genes are subfunctionalized with respect to their expression, and are thus good candidates for the model of subfunctionalized genes. For ray-finned fishes, a reliable phylogenetic framework, which is essential for comparative evolutionary analyses, is available due to recent progress in molecular phylogenetic studies [16-21]. In addition, the basal lineages of ray-finned fishes, including Semionotiformes (gar fish) and Amiiformes (amia), have only one *Pgi *locus [12]. This single-copy gene may be the direct descendant of the ancestral unduplicated *Pgi *in ray-finned fishes, and thus can be considered an appropriate outgroup gene for comparison between the duplicated *Pgi *genes.

The *Pgi-1 *and *Pgi-2 *genes, which were expected to be subfunctionalized in their expression, also differ in the net electric charge of their encoded proteins [12,13,15]. The electric charge of soluble proteins such as PGIs is a structural property brought by a large number of multiple amino acid residues and is involved in the adaptive evolution of several soluble proteins [e.g., [22-24]], such as an acquisition of protein thermostability [25-27]. Therefore, the evolution of electric charge in the duplicated PGI proteins is an interesting subject to investigate regarding the evolution of novel protein properties after subfunctionalization.

In this study, we examined first whether the spatial expression patterns of duplicated *Pgi *genes in ray-finned fishes are compatible with predictions based on the subfunctionalization model of duplicate gene evolution. Next, by focusing on the electric charges of the PGI proteins, we analyzed the underlying evolutionary process producing novel protein properties after gene duplication through ancestral sequence inference using the maximum likelihood (ML) method based on a reliable phylogenetic framework of ray-finned fishes, and also using three-dimensional (3-D) structural information on the protein.

## Results

### Duplication and subfunctionalization of the *Pgi *genes in teleost fishes

**Figure 1:**
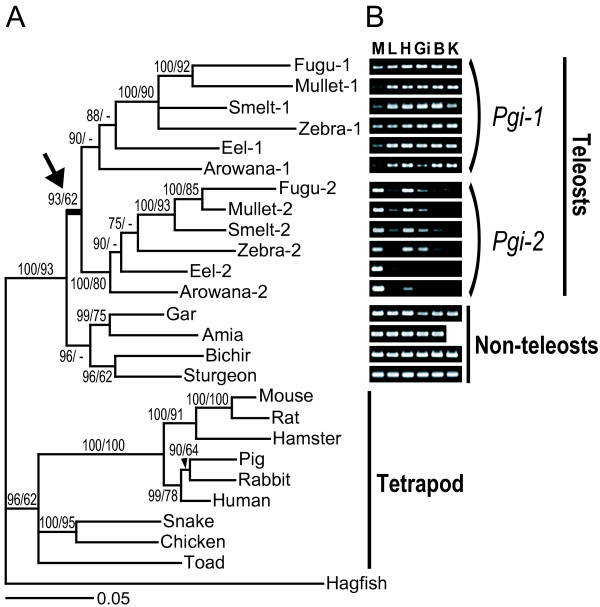
**Molecular phylogeny and spatial expression patterns of *Pgi***. (A) Bayesian tree of *Pgi *genes derived from 20 vertebrates. Numbers indicate percent posterior probabilities for the Bayesian tree (left) and bootstrap support values by the maximum likelihood method (right). Arrow denotes a gene duplication event. In cDNA clones, only one *Pgi *was identified from non-teleosts, whereas two *Pgi *genes were identified from teleosts. The two *Pgi *genes differed by about 20% in amino acid sequence, and were grouped into separate clades (*Pgi-1 *and *Pgi-2*). In both clades, the gene relationships were consistent with the evolutionary relationships of teleost species [18, 19, 21]. (B) Partial-length gel images of the RT-PCR expression analysis of *Pgi *genes and positive control (*β-actin*) genes in ray-finned fishes. The tree in the left panel shows the relationships among the *Pgi *genes inferred in this study. The black circle on the tree denotes the timing of the *Pgi *gene duplication event. Letters indicate tissues: M, muscle; L, liver; H, heart; Gi, gill; B, brain; K, kidney. Full-length gels, including negative controls and size markers, are presented in Additional file 1: Fig. S5.

Our molecular phylogenetic analyses of vertebrate *Pgi *genes showed that the *Pgi-1 *and *Pgi-2 *genes in teleost fishes resulted from a gene duplication event that occurred before the radiation of teleosts but after the separation of basal non-teleost ray-finned fishes (Figure 1A, arrow). The *Pgi *duplication appears to have derived from the ancient teleost genome duplication [28-30] because the phylogenetic position of the *Pgi *duplication confirmed here is the same as that of the estimated teleost-specific genome duplication event [31,32], and gene content around the *Pgi *locus in the human genome is partly conserved in the corresponding regions of both zebrafish *Pgi *loci (on chromosomes 13 and 25) [see Additional file 1: Fig. S1], which rules out the possibility of a tandem or single-gene duplication of the *Pgi *in teleosts. This condition allows us to study the duplicated *Pgi *genes without considering interlocus concerted evolution, which complicates the analysis of functional divergence in duplicated genes.

A reverse transcriptase-polymerase chain reaction (RT-PCR)-based expression analysis showed that the *Pgi *gene in non-teleost ray-finned fishes was expressed in all tissues examined (Figure 1B), confirming that this gene is the direct descendant of the ancestral unduplicated *Pgi *with no tissue specificity, as in tetrapods [12,13,15]. In contrast, the teleost *Pgi-1 *gene was expressed mainly in internal organs, including the liver, heart, gill, brain, and kidney, and weakly in the muscle, whereas *Pgi-2 *was expressed mainly in the heart and muscle. The differential expression patterns of *Pgi-1 *and *Pgi-2 *support the concept of subfunctionalization [2,3], which is the complementary loss of subsets in the expression organs of the ancestral gene. Thus, the *Pgi *gene family in ray-finned fishes is an appropriate model for studying molecular evolution after subfunctionalization.

### Evolution of the electric charges of duplicated PGI proteins in teleost fishes

The *Pgi-1 *and *Pgi-2 *genes in teleosts, which are subfunctionalized with respect to their expression, differed significantly in the predicted electric charge of their encoded proteins (*P *= 0.0040, Mann-Whitney *U *test, *n *= 12; see Table 1). The estimated isoelectric points (pI) of PGI-1 were 6.21–6.36 (average, 6.31), and those of PGI-2 were 6.75–7.36 (average, 7.17), with no overlap. In contrast to this clear difference, the PGI enzyme active sites in both isoforms (Ile156, Gly158, Ser159, Ala208, Ser209, Loop 210–214, Thr217, Arg272, Gln353, Glu357, His388, Gln511, Helix 512–520, and Lys518; [33]) were totally conserved among all fishes and tetrapods examined [see Additional file 1: Fig. S2]. Moreover, no significant difference was observed in peptide length or predicted overall hydrophobicity between the two isoforms [see Table 1]. The estimated pI values for the ancestral PGI in non-teleosts were intermediate (6.62–6.84; average, 6.78).

**Table 1:** Biochemical parameters of vertebrate PGI proteins.

	Biochemical characters^1^			No. of hydrophilic charged residues		
		
	No. of amino acids	Hydro-phobicity (GRAVY)	Isoelectric point (pI)	Positively charged (Arg+Lys)	Negatively charged (Asp+Glu)	Difference^2^
Teleost fish PGI-1						
Fugu-1	552	-0.261	6.33	58	64	-6
Mullet-1	553	-0.282	6.30	57	63	-6
Smelt-1	553	-0.284	6.21	54	62	-8
Zebrafish-1	553	-0.265	6.45	53	58	-5
Eel-1	553	-0.359	6.36	57	63	-6
Arowana-1	553	-0.277	6.22	56	64	-8
						
Teleost fish PGI-2						
Fugu-2	553	-0.294	6.96	59	61	-2
Mullet-2	553	-0.265	7.85	61	60	1
Smelt-2	552	-0.337	7.36	64	64	0
Zebrafish-2	553	-0.304	6.82	59	61	-2
Eel-2	553	-0.285	6.75	58	61	-3
Arowana-2	553	-0.280	7.07	63	64	-1
						
Non-teleost fish PGI						
Sturgeon	555	-0.356	6.82	59	61	-2
Gar	555	-0.308	6.83	59	61	-2
Amia	555	-0.355	6.62	60	63	-3
Bichir	556	-0.278	6.84	58	60	-2
						
Tetrapod PGI						
Toad	553	-0.226	7.68	59	58	1
Snake	553	-0.237	8.72	62	57	5
Chicken	553	-0.255	8.34	64	61	3
Mouse	558	-0.294	7.75	61	60	1
Pig	558	-0.340	7.79	62	61	1
Rat	558	-0.285	7.38	61	61	0
Hamster	558	-0.322	7.08	59	60	-1
Rabbit	558	-0.292	7.11	58	59	-1
Human	558	-0.344	8.42	62	59	3
						
Jawless fish PGI						
Hagfish	554	-0.238	7.82	57	56	1

^1^The predicted overall hydrophobicity (GRAVY; grand average of hydropathicity) and pI values of the PGI proteins were estimated based on the amino acid sequences translated from the cDNA sequences of *Pgi *genes using the ProtParam tool [49]

^2^Differences in number of positively and negatively charged residues in each PGI protein

**Figure 2:**
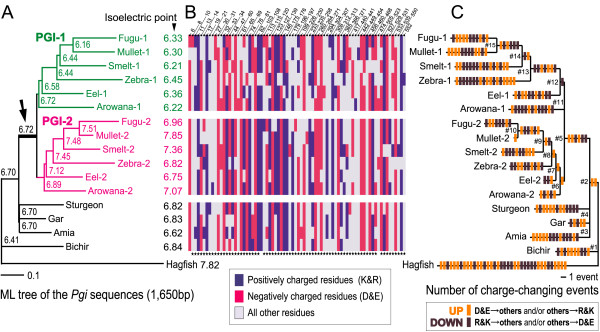
**Current states and inferred evolutionary process of electric charge change in PGI isoforms**. (A) Maximum likelihood tree of *Pgi *genes in ray-finned fishes inferred by BASEML [34] with a known phylogeny [18]. Numbers indicate estimated pI. Arrow denotes a gene duplication event. (B) Amino acid sites that differ by the presence or absence of hydrophilic charged residues between current PGI-1 and PGI-2. Positively charged residues are blue; negatively charged residues, red; other residues, light gray. The numbers above refer to the amino acid positions of PGI [33]. The stars below indicate sites located on the molecular surface. (C) Inferred charge-changing substitution events mapped over the PGI phylogeny. Orange and brown bars denote upward and downward charge changes, respectively.

Between PGI-1 and PGI-2, 76 amino acid sites differed by the presence or absence of hydrophilic charged residues [Lys (K), Arg (R), Asp (D), and Glu (E)], which mainly contribute to net protein charge (Figure 2B). These sites were not fixed for a unique charge state in the examined PGI-1 or PGI-2 proteins, except at position 294 (Gln in PGI-1 and Lys in PGI-2). Furthermore, only a few unique charged sites were shared among two or more genealogically related isoforms: five in PGI-1 (positions 27, 61, 78, 199, and 454) and two in PGI-2 (positions 17 and 226). These observations imply that very few specific amino acid residues were acquired in the early ancestral proteins and involved in the differences in electric charges between current PGI-1 and PGI-2.

The underlying process of the electric charge evolution of the PGI proteins can be inferred by ML sequence reconstructions [34] based on the recent ray-finned fish phylogeny [18]. We applied this approach and show the results in Figure 2A. Our results suggest that after gene duplication, pI values in the PGI-1 clade gradually decreased, whereas those in the PGI-2 clade increased. Next, we assigned charge-changing substitutions (between Lys/Arg or Asp/Glu and other residues) to the tree branches based on pairwise sequence comparisons among the inferred ancestral sequences or between the extant and ancestral sequences along the tree topology. This result of assignment (Figure 2C) showed that the charge-changing substitutions were inferred to have occurred in excess (5–28) of that expected (3–5) for parsimonious evolution in electric charge differences between PGI-1 and PGI-2 (Figure 2C; see also Table 1). Figure 2C also shows that the charge-changing substitutions have occurred in both directions (either upward or downward) on most branches at various amino acid sites (76 sites shown in Figure 2B) [see Additional file 1: Table S1]. An analysis using parsimony yielded similar results [see Additional file 1: Fig. S3].

### Statistical analyses of the spatial clustering of inferred amino acid substitutions

**Figure 3:**
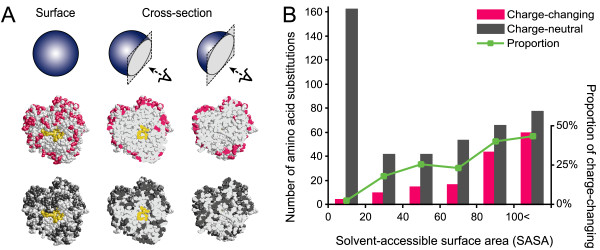
**Spatial locations of inferred amino acid substitutions in the PGI structure**. (A) Maximum likelihood-inferred charge-changing substitution sites after the *Pgi *duplication are colored magenta; charge-neutral substitution sites, dark gray; enzyme active sites, yellow. Full molecular models are shown on the left, and two cross sections are shown center and right. The inferred charge-changing sites localize to the surface of the PGI molecule (73 charge-changing sites/234 total surface sites, 3 charge-changing sites/316 total interior sites; *P *= 0.0000, two-tailed Fisher's exact test), in contrast to the inferred charge-neutral sites (106 charge-neutral sites/234 total surface sites, 183 charge-neutral sites/316 total interior sites; *P *= 0.1040, two-tailed Fisher's exact test) (B) Histograms of the inferred number of charge-changing and charge-neutral substitutions after the *Pgi *duplication. The solid green line denotes the proportion of charge-changing substitutions per total substitutions within the site classes based on solvent accessibility (horizontal axis): this proportion significantly increases with solvent-accessible surface area (*P *= 0.0000, Cochran – Armitage trend test, *n *= 584).

Based on 3-D structural information on the PGI protein molecule, we further examined whether the inferred charge-changing substitutions were actually involved in the evolution of electric charge. The results of this analysis on the inferred substitution sites and number of substitutions are shown in Figure 3A and 3B, respectively. Figure 3A shows that the inferred charge-changing substitution sites after the *Pgi *gene duplication (colored in magenta) were concentrated at the surface of the PGI molecule, in contrast to the inferred charge-neutral substitution sites (colored in dark gray) that contribute little or nothing to net protein charge. The inferred number of charge-changing and charge-neutral substitutions that can potentially occur at identical sites also followed the same trend (Figure 3B).

However, because water-soluble proteins such as PGI are generally surrounded by a hydrophilic shell containing a high density of polar residues, it is natural to expect the charge-changing substitutions to occur more frequently on the surface without any selection. Considering this expected mutation bias, further analysis was performed (Table 2; for details, see Methods). This comparison of theoretically expected and ML-inferred numbers of charge-changing and charge-neutral substitutions imply that charge-neutral substitutions have occurred more frequently than expected at the molecular surface [ML-inferred value, 63.1% = 277/(162 + 277); expected value, 55.5% = 230.17/(184.46 + 230.17)], consistent with the general observation that molecular evolutionary rates are faster at the surface than in the interior portions of water-soluble proteins [35,36]. However, what is most important in this table is that the proportion of charge-changing substitutions concentrated at the surface of the PGI molecule is much greater than that expected by chance [ML-inferred value, 97.2% = 141/(4 + 141); expected value, 78.8% = 133.45/(35.92 + 133.45)]. These charge-changing substitutions do not appear to be derived from differential neutral evolution of base composition or codon usage between *Pgi-1 *and *Pgi-2 *genes, as demonstrated by the fact that GC content and codon usage frequencies are not significantly different between *Pgi-1 *and *Pgi-2 *(GC content: *P *= 0.0782, Mann-Whitney *U *test, *n *= 12; rank order of codon usage: *r*_s _= 0.9509, *n *= 64).

**Table 2:** Analytically inferred and theoretically predicted numbers of charge-changing and charge-neutral substitutions.

	Charge-changing		Charge-neutral		Sum
			
	Interior	Surface	Interior	Surface	
Maximum likelihood-inferred numbers	4	141	162	277	584
Theoretical prediction	35.92	133.45	184.46	230.17	584.00
*P *value*	0.00004		0.00150		

* *P *values are from two-tailed exact tests.

## Discussion

The results of phylogenetic analysis, RT-PCR-based expression analysis, and sequence comparison of *Pgi *genes in teleost fishes suggest that after subfunctionalization of the duplicated *Pgi *genes in the ancestor of teleost fishes, the electric charges of the PGI-1 and PGI-2 proteins diverged. This evolution can be interpreted according to the sub-neofunctionalization model of gene evolution [2-5], which proposes that the partitioning of function between the duplicated genes alters the selective environment at each locus, resulting in structural fine-tuning or adaptation of the encoded proteins by positive selection. That is, the divergent evolution of the electric charges in the duplicated PGI isoforms is the consequence of specialization for the specific function (glycolysis or gluconeogenesis) or distinct cellular environment of tissues where each isoform is predominantly expressed (see Figure 1B), as suggested for other water-soluble proteins [22-24].

The present comparative evolutionary analysis implies that since the gene duplication event, the electric charges of the two PGI isoforms changed steadily through many charge-changing substitutions in both directions of charge change; only a few charged amino acid sites were specific to PGI-1 or PGI-2 (Figure 2). Such charge-changing substitutions concentrated at the surface of PGI molecule (Figure 3) were inferred to have occurred much more frequently than expected in the parsimonious evolution of electric charge difference between the two isoforms (see Figure 2C and Table 1). From these observations, two possible scenarios are proposed for the evolution of protein charge in duplicated PGI isoforms: protein charges in PGI-1 decreased gradually, while those in PGI-2 increased; alternatively, charge divergence between PGI-1 and PGI-2 was completed soon after the duplication and before the radiation of teleosts, followed by maintenance of the protein charges under purifying selection, while stochastic charge-changing substitutions by drift occurred among lineages. In either scenario, we can conclude that the surface charge evolution of PGI proteins was not driven by strong selection on individual amino acid sites leading to permanent fixation of a particular residue, but rather was driven by weak selection on a large number of amino acid sites and consequently by steady directional or purifying selection on the overall structural properties of the protein, which is derived from many modifiable sites. This mode of molecular evolution agrees with the understanding that most proteins are substantially tolerant of a broad spectrum of substitutions and thus may harbor many amino acid sites available for evolutionary modification [37]. Our study provides the first plausible evidence of adaptive protein evolution through such selection.

The mode of molecular evolution proposed in this study would be difficult to find using existing methods that detect strong selection for particular substitutions. We applied such an analysis to identify positively selected sites after *Pgi *gene duplication using DIVERGE version 1.04 [38]; however, the results were not clear (data not shown). Further analysis using the program CODEML [34] did not detect the acceleration of the rate of nonsynonymous substitution, not showing selection for amino acid changes (estimated ω was 0.01 to 0.23). Even if, in general, a significant excess of amino acid change is detected, such methodology itself cannot rule out possible confounding effects, or alternative interpretations, particularly the relax of purifying selection [39]. In a previous study, an analysis using the program HonNew [40] also failed to detect selection for charge-changing substitutions in teleost PGIs, leading to the conclusion that the charge change in the duplicate PGIs of teleosts may be selectively neutral [13]. The mode of molecular evolution presented here, in which diverse evolutionary resolutions exist at the level of a primary sequence that corresponds to a certain selective pressure on a protein property, may be relevant to various cases of adaptive modification in proteins, such as hydrophobic properties, molecular size, and electric charge. This may be an important pathway underlying physiological adaptation, along with protein evolution by simple amino-acid changes, gene deletion or silencing, and possibly *cis*-regulatory changes [39,41].

## Conclusion

In this paper, we provide the evidence that relatively weak selection on a large number of amino acid sites drives the evolution of novel charge-state of duplicated phosphoglucose isomerases, which are subfunctionalized in teleost fishes. Such mode of adaptive molecular evolution, which was hardly recognizable by existing analytical methods aiming to detect strong selection on individual amino acid changes, may play a substantial role in the evolution of novel proteins.

## Methods

### Taxonomic sampling

Our data set contains representatives from divergent lineages of ray-finned fishes, as follows – basal non-teleost ray-finned fishes: *Polypterus ornatipinnis *(bichir), *Acipenser ruthenus *(sturgeon), *Amia calva *(amia), and *Lepisosteus osseus *(gar); teleosts: *Osteoglossum bicirrhosum *(arowana) and *Anguilla anguilla *(eel) from basal groups, *Plecoglossus altivelis *(smelt) and *Danio rerio *(zebrafish) from intermediate groups, and *Mugil cephalus *(mullet) and *Fugu rubripes *(fugu) from derived groups. Live specimens, which were obtained either from local shops or other investigators in Japan, were treated according to the ethical recommendations of the Ichthyological Society of Japan and the University of Tokyo.

### Cloning and sequencing

Total RNA was extracted from fresh skeletal muscle and liver tissue using TRIzol reagent (Invitrogen) and reverse-transcribed into first-strand cDNA with oligo-dT adaptor primer using an RNA PCR kit (TaKaRa). Partial *Pgi *cDNA was amplified using PCR with vertebrate universal degenerate primers [13]. The well amplified DNA fragments were purified using a MinElute gel extraction kit (Qiagen), ligated into the pGEM-T Easy Vector system (Promega), transmitted into competent *E. coli *(Competent High DH5a, Toyobo), and sequenced with an ABI PRISM 3100 (Applied Biosystems) using T7 or SP6 primers. The partial *Pgi *sequences were used to design gene-specific primers (GSPs) for RACE PCR [see Additional file 1: Table S2]; 3' RACE PCR was conducted with the sense GSP and M13 primer M4 (TaKaRa) and the first-strand cDNA as the template. For 5' RACE, double-stranded cDNA PCR libraries were synthesized from 1 μg of total RNA using the cDNA synthesis kit (M-MLV version; TaKaRa) combined with the cDNA PCR library kit (TaKaRa). Then, 5' RACE PCR was conducted with the antisense GSP and CA primer (TaKaRa). Subcloning and sequencing were performed as above.

### Phylogenetic analysis

The *Pgi *genes from 20 vertebrates were phylogenetically analyzed with the Bayesian and ML methods using the programs MrBayes 3.0B4 [42] and PAUP 4.0b10 [43], respectively. The species used [GenBank accession numbers or Ensembl Transcript IDs of the *Pgi *gene(s)] were as follows: bichir (AB282684*), sturgeon (AB282688*), amia (AB282681*), gar (AB282687*), arowana (*Pgi-1*: AB282682* and *Pgi-2*: AB282683*), eel (*Pgi-1*: AB282685* and *Pgi-2*: AB282686*), smelt (*Pgi-1*: AB282690* and *Pgi-2*: AB282691*), zebrafish (*Pgi-1*: AJ306395 and *Pgi-2*: AJ306396), mullet (*Pgi-1*: AJ306392 and *Pgi-2*: AJ306393), fugu (*Pgi-1*: NEWSINFRUT00000145974 and AB282689*, and *Pgi-2*: NEWSINFRUT00000159975), *Homo sapiens *(human; K03515), *Sus scrofa *(pig; X07382), *Oryctolagus cuniculus *(rabbit; AF199601), *Cricetulus griseus *(hamster; Z37977), *Mus musculus *(mouse; M1422), *Rattus norvegicus *(rat; ENSRNOT00000032613), *Gallus gallus *(chicken; ENSGALT00000007948), *Boiga kraepelini *(snake; AJ306394), *Bufo melanostictus *(toad; AJ306397), and *Paramyxine yangi *(hagfish; AJ306391). Newly cloned sequences in this study (marked with asterisks) were named under the denomination of PGI isozymes [12]. Bayesian and ML trees were constructed under the GTR + I + Γ model [44], which was selected as the best-fitting model of nucleotide substitution by hierarchical likelihood ratio tests (hLRTs) [45,46] with 1100 base pairs (bp) of the *Pgi *coding region (excluding the third codon position) [see Additional file 1: Fig. S4]. The Bayesian posterior probabilities of the phylogeny and its branches were determined from 9901 trees. Support for heuristic ML analysis was assessed using 100 bootstrap replications.

### Synteny analysis

The genomic regions around the *Pgi *locus (or loci) in the human, chicken, and zebrafish genomes were investigated and compared. Genomic data from the pufferfishes *Fugu rubripes *and *Tetraodon nigroviridis *were not useful in this analysis because the locations of their *Pgi *loci were not determined. Data on the neighborhood of the *Pgi *locus in the human and chicken genomes were obtained from the NCBI Mapviewer Web site [47]. Twenty-seven protein-coding genes were identified around the human PGI locus, within a 1.8-Mb-long region on chromosome 19. The nucleotide sequences of these human genes were subjected to BLASTN searches against the zebrafish genome sequences using the Ensembl BLASTN search service [48]. The matches detected with an *E*-value threshold of <10^-3 ^were checked visually. Then, we selected identifiable genes described as putative orthologs of the queries. Their genomic location data were used to rebuild the synteny maps around the zebrafish *Pgi *loci.

### Gene expression analysis

RT-PCR was performed for expression analysis of the *Pgi *genes. The primers used are described in [see Additional file 1: Table S3]. They were designed as follows: to distinguish the duplicate *Pgi *loci in teleosts, the 3' region of one primer from each primer pair was made to locate the differential nucleotide site between the two loci of the species concerned, and to avoid false amplification from genomic DNA contaminants, each primer pair was designed to span a *Pgi *exon/intron boundary considered conservative among vertebrates. Total RNA was extracted from liver, skeletal muscle, heart, gill filament, brain, and kidney (or gonad) tissues of fresh fish samples. RNA extraction, reverse-transcription into first-strand cDNA and PCR were performed in the same manner as mentioned in the *Cloning and sequencing *section. The thermal-cycle profile was as follows: 1 cycle at 94°C for 2 min; 30 cycles at 94°C for 30 sec, 60°C for 30 sec, and 72°C for 30 sec; followed by 1 cycle at 72°C for 7 min. As a positive control for gene expression, *β-actin *cDNA was amplified using the primers 5'-GACATGGAGAAGATCTGGCA-3' and 5'-TGATCCACATCTGCTGGAAGGT-3' (predicted product size = 834 bp), which were designed by Dr. Kaoru Kuriiwa of the National Museum of Nature and Science, Tokyo. These primer sequences were based on a highly conserved region of the *β-actin *gene in mangrove killifish, *Rivulus marmoratus *(GenBank accession number AF168615). The amplified DNA fragments were separated on a 2.0% L03 agarose gel (TaKaRa), stained with ethidium bromide, and visualized under UV light. GeneRuler™ 100 bp DNA Ladder Plus (MBI Fermentas) was used as a size marker for electrophoresis.

### Charge evolution analysis

The ML inference of the ancestral sequences of *Pgi *genes was performed by BASEML [34] based on the phylogeny of ray-finned fishes using whole mitochondrial genome data [18]. Tetrapods were excluded from this analysis because of their absence in this tree. Nucleotide sequence alignments of the coding region of *Pgi *cDNAs (1650 bp, without ambiguous regions) from 10 ray-finned fishes plus hagfish were used. The GTR + Γ [44] model was selected as the best fitting model by the hLRTs [see Additional file 1: Table S4]. The average overall accuracy of the reconstructed sequences (#1–#15) [see Additional file 1: Appendix] was 0.948 ± 0.003 SE. The pI values were estimated from the deduced amino acid sequences using the ProtParam tool [49]. The solvent-accessible surface area (SASA) of each amino acid residue was estimated with GETAREA 1.1 [50] for the dimeric PGI protein structure using a solvent radius of 1.4 Å (approximately the size of a water molecule). Rabbit PGI [PDB: 1XTB] [33] was used as a reference structure. The structural portion of the PGI composed of amino acid residues with more than 20 Å ^2 ^SASA was considered "molecular surface." This boundary mostly agrees with other criteria based on the ratio of side-chain surface area to random coil value per residue [50]. A three-dimensional graphical model of the PGI molecule was constructed using RasMol [51].

### Calculation of the expected spatial distribution of amino acid substitutions

To determine which model of amino acid substitution provided the best fit to the data (550-amino-acid sequence of PGIs from 11 fishes and the known phylogenetic framework of ray-finned fishes [18]), likelihood ratio tests were conducted among pairs of five models mounted in PAML 3.13d [34]. Parameters F and Γ were incorporated in this analysis. As a result, the amino acid substitution matrix JTT [52] gave the highest likelihood score (lnL = -5851.41); the second-best matrix was Dayhoff [53] (lnL = -5865.41). Using the JTT matrix (*m*_*ij*_), transition rates between pairs of amino acids (*P*_*ij*_) were calculated by the equation

Pij=mijfiμi∑j=AlaValmij,(i≠j)
 MathType@MTEF@5@5@+=feaafiart1ev1aaatCvAUfKttLearuWrP9MDH5MBPbIqV92AaeXatLxBI9gBaebbnrfifHhDYfgasaacH8akY=wiFfYdH8Gipec8Eeeu0xXdbba9frFj0=OqFfea0dXdd9vqai=hGuQ8kuc9pgc9s8qqaq=dirpe0xb9q8qiLsFr0=vr0=vr0dc8meaabaqaciaacaGaaeqabaqabeGadaaakeaafaqabeqacaaabaGaemiuaa1aaSbaaSqaaiabdMgaPjabdQgaQbqabaGccqGH9aqpdaWcaaqaaiabd2gaTnaaBaaaleaacqWGPbqAcqWGQbGAaeqaaOGaemOzay2aaSbaaSqaaiabdMgaPbqabaacciGccqWF8oqBdaWgaaWcbaGaemyAaKgabeaaaOqaamaaqadabaGaemyBa02aaSbaaSqaaiabdMgaPjabdQgaQbqabaaabaGaemOAaOMaeyypa0JaeeyqaeKaeeiBaWMaeeyyaegabaGaeeOvayLaeeyyaeMaeeiBaWganiabggHiLdaaaOGaeiilaWcabaGaeiikaGIaemyAaKMaeyiyIKRaemOAaOMaeiykaKcaaaaa@53B3@

where *f*_*i *_is the normalized frequency and μ_*i *_is the relative mutability of each amino acid. The parameter *f*_*i *_was estimated separately for the surface and interior portions of the inferred common ancestral protein of PGI-1 and PGI-2 (node #5 in Figure 2C) to consider differential amino acid composition in different parts of the protein [see Additional file 1: Table S5]. Based on the resultant *P*_*ij*_, we estimated the theoretical ratio of the charge-changing substitutions to charge-neutral substitutions (Σ*P*_charge-changing_: Σ*P*_charge-neutral_) of the surface (*r*_1_:*r*_2_) and interior (*r*_3_:*r*_4_) portions of the PGI protein molecule under the assumption of random mutation.

According to the null hypothesis that all pairs of amino acid substitutions occur regardless of their spatial locations, the amino acid substitution events would be spatially distributed into the surface and interior portions of the PGI protein along the ratio of the numbers of amino acid substitution sites at the surface (132 sites) to the interior (80 sites) of the PGI protein since their gene duplication. Accounting for the spatial-differential amino acid composition as described above, the expected spatial distribution of amino acid substitutions shown in Table 2 was estimated based on the ratio of charge-changing substitutions in the molecular surface:charge-neutral substitutions in the molecular surface:charge-changing substitutions in the molecular interior:charge-neutral substitutions in the molecular interior = 132*r*_1_:132*r*_2_:80*r*_3_:80*r*_4_.

## Authors' contributions

YS and MN designed the study. YS carried out the molecular work and the analyses, and drafted the manuscript. MN participated in coordination and helped to draft the manuscript. Both authors read and approved the final version of the manuscript.

## Supplementary Material

Additional file 1 Supplementary material files. This PDF file includes supplementary tables S1 – S5, figures S1 – S5, and appendix (inferred ancestral nucleotide sequences of *Pgi *genes using maximum likelihood methods). Click here for file
