# Supplementary material for: Post-duplication charge evolution of phosphoglucose isomerases in teleost fishes through weak selection on many amino acid sites
Source: BMC Evol Biol. 2007 Oct 29;7:204. doi: 10.1186/1471-2148-7-204 (PMC2176064; doi:10.1186/1471-2148-7-204)
Supplement: Additional file 1 — Supplementary material files. This PDF file includes supplementary tables S1 – S5, figures S1 – S5, and appendix (inferred ancestral nucleotide sequences of Pgi genes using maximum likelihood methods). [file 1471-2148-7-204-S1.pdf]

Additional file 1 for

Post-duplication charge evolution of phosphoglucose isomerases in teleost fishes through weak selection on many amino acid sites

Yukuto Sato[1,2§], Mutsumi Nishida [1§]

[1]Division of Molecular Marine Biology, Ocean Research Institute, The University of Tokyo, 1-15-1 Minamidai, Nakano-ku, Tokyo 164-8639, Japan
[2]Department of Aquatic Bioscience, Graduate School of Agricultural and Life Sciences, The University of Tokyo, 1-1-1 Yayoi, Bunkyo-ku, Tokyo 113-8657, Japan

[§]Corresponding authors

Email addresses:
YS: ysato@ori.u-tokyo.ac.jp
MN: mnishida@ori.u-tokyo.ac.jp

This PDF file includes supplementary tables S1—S5, figures S1—S5, and appendix

Supplementary Tables

Table S1. Numbers and details of the charge-changing substitutions estimated to have occurred between nodes of the *Pgi* gene tree.

	Direction of electric charge change[1]	
	Up	Down
node#1 → node#2	2	0
	T→K (231), E→Q (529)	
node#2 → node#3	0	0
node#2 → node#5	3	3
	E→A (78), N→K (123), G→K (233),	K→T (231), N→D (260), K→Q (366)
node#3 → node#4	0	0
node#3 → Amia	7	7
	Q→K (10), Q→K (14), E→Q (34), E→L (77), T→K (85), Q→K (294), E→Q (468)	H→E (111), N→D (199), K→A (235), K→Q (313), T→D (368), R→Q (445), K→T (479)
node#4 → Gar	3	2
	E→G (34), D→G (113), Q→R (294)	K→Q (33), K→N (44)
node#4 → Sturgeon	7	6
	E→Q (13), N→K (19), N→K (24), E→Q (30), E→Q (78), D→A (113), N→K (123)	A→D (18), K→Q (88), K→S (231), N→D (371), K→N (460), K→Q (523)
node#5 → node#11	0	0
node#11 → node#12	0	1
		A→D (531)
node#12 → node#13	3	4

	D→A (69), E→K (197), N→K (365)	K→E (33), K→Q (44), R→N (179), Q→D (366)
node#13 → node#14	1	1
	S→K (454)	A→E (78)
node#14→ node#15	2	4
	E→Q (13), E→Q (61)	Q→E (27), K→E (65), K→T (175), N→D (199)
node#11 → Arowana-1	4	9
	Q→R (10), D→G (113), E→G (197), T→K (440)	A→D (31), K→N (44), N→D (60), K→G (63), K→T (175), K→T (233), K→P (235), K→S (313), N→D (474)
node#12 → Eel-1	5	6
	Q→K (10), D→A (69), D→G (113), D→N (258), S→R (308)	K→N (44), K→T (115), K→Q (146), G→D (271), K→A (313), N→D (417)
node#13 → Zebra-1	9	9
	E→A (33), E→A (139), D→N (260), E→G (312), D→S (366), E→Q (449), E→Q (468), D→N (505), F→R (552)	K→S (65), A→D (69), R→V (74), R→Q (82), R→W (103), K→Q (127), K→Q (197), K→Q (313), N→D (417)
node#14 → Smelt-1	7	9
	N→K (19), D→A (34), D→N (260), E→A (442), D→P (456), E→Q (468), D→S (531)	Q→E (44), K→Q (65), K→S (115), K→E (175), K→N (233), R→H (307), N→D (417), K→A (454), K→N (460)
node#15 → Mullet-1	7	5
	D→H (6), E→K (33), E→A (65), N→K (179), T→K (441), D→G (456), D→N (505)	K→Q (197), K→G (233), K→Q (313), N→D (371), K→E (460)
node#15 → Fugu-1	6	5

	Q→R (10), E→A (30), D→K (32), E→Q (81), N→K (304), E→A (533)	N→E (8), T→E (108), K→T (115), K→S (233), K→S (313)
node#5 → node#6	1	0
	Q→K (294)	
node#6 → node#7	1	0
	E→Q (468)	
node#7 → node#8	1	0
	Q→K (14)	
node#8 → node#9	1	1
	E→Q (197)	N→D (417)
node#9 → node#10	3	3
	E→Q (13), E→A (226), Q→K (529)	K→N (14), K→T (17), Q→E (230)
node#6 → Arowana-2	3	2
	G→R (21), N→K (365), Q→K (529)	K→S (313), N→D (417)
node#7 → Eel-2	1	2
	E→G (264),	K→H (65), K→P (235)
node#8 → Zebra-2	4	6
	D→T (34), D→V (47), Q→K (366), E→Q (533)	K→N (11), A→E (31), K→A (235), K→Q (313), N→D (474), K→N (550)
node#9 → Smelt-2	5	5
	E→K (13), N→K (365), E→Q (527), A→K (531), N→K (535)	K→E (14), A→D (31), G→D (176), H→D (366), K→Q (523)
node#10 → Mullet-2	1	0
	D→G (113)	
node#10 → Fugu-2	3	5

	T→K (108), E→G (120), E→Q (230)	N→D (14), K→N (123), R→H (179), A→E (196), K→S (235)

[1]Amino acid substitutions assumed to increase ('Up') and decrease ('Down') the electric charge of the protein.

Table S2. Gene-specific primers used for cDNA cloning or partial amplification of *Pgi* in this study.

Primers	Purposes	Sequence (5'→3')[1]
For bichir (*Polypterus ornatipinnis*)		
PoorPGI-3'-1	3'-RACE of *Pgi*	GGC CAA CAA ATT CAA TGG TC
PoorPGI-3'-2	3'-RACE of *Pgi*	CCT TCA TTT TAG GTG CAC TGA
PoorPGI-5'-1	5'-RACE of *Pgi*	CAG TGG TGT ATT GTG GAA GTG
For sturgeon (*Acipenser ruthenus*)		
AcruPGI-3'-1	3'-RACE of *Pgi*	GAG CAC AAG ATC TTC GTA CAG G
AcruPGI-5'-1	5'-RACE of *Pgi*	GCT CCA CTC AGA AGA TGC TC
AcruPGI-5'-2	5'-RACE of *Pgi*	ACA GGG ACA TTY TTW TCC AAG
For amia (*Amia calva*)		
AmcaPGI-3'-1	3'-RACE of *Pgi*	AAC TGC TGC CTC ATA AGG TC
AmcaPGI-3'-2	3'-RACE of *Pgi*	TCT TCA CGA AAC TGA ATC CC
AmcaPGI-5'-1	5'-RACE of *Pgi*	CGG AAA TGA TTG TCC ATC CAG T
For gar (*Lepisosteus osseus*)		
LeosPGI-3'-1	3'-RACE of *Pgi*	TGA TTG CTA TGT ATG AAC AC
LeosPGI-3'-2	3'-RACE of *Pgi*	ATC GGC ATT GGT GGA TCT G
LeosPGI-5'-1	5'-RACE of *Pgi*	ATG GAT GAG CTG GTA GAA GG
For arowana (*Osteoglossum bicirrhosum*)		

OsbiPGI1-3'-1	3'-RACE of *Pgi-1*	CAC AAA GTG TTT GAG GGA A
OsbiPGI1-5'-1	5'-RACE of *Pgi-1*	AAG GCA TGA GTC TCT GCT T
OsbiPGI2-3'-1	3'-RACE of *Pgi-2*	AAT TTG CAC CAC AAG ATC C
OsbiPGI2-3'-2	3'-RACE of *Pgi-2*	ACA TAG GCT TTG AGA ACT T
OsbiPGI2-5'-1	5'-RACE of *Pgi-2*	TGG AAG AAG TTG ATG TAC CAG
For eel (*Anguilla anguilla*)		
AgagPGI1-3'-1	3'-RACE of *Pgi-1*	GTT CAA GAA GTT GAC CCC TTT C
AgagPGI1-5'-1	5'-RACE of *Pgi-1*	TGG AAG AAG TTG ATG TAC CAG A
AgagPGI2-3'-1	3'-RACE of *Pgi-2*	AGA AGC TGA CAC CAT TCA TCC
AgagPGI2-5'-1	5'-RACE of *Pgi-2*	AGT GGT TGT CCA TCC AGT GAG
For smelt (*Plecoglossus altivelis*)		
PlalPGI1-3'-1	3'-RACE of *Pgi-1*	TCG CTG CAT ACT TCC AAC AG
PlalPGI1-5'-1	5'-RACE of *Pgi-1*	TGT CTC TGA TAG GGT GTT GGG
PlalPGI2-3'-1	3'-RACE of *Pgi-2*	ACC AAG GTA CTC GCA TGG TC
PlalPGI2-5'-1	5'-RACE of *Pgi-2*	TCC TTC TTA GCC TCC TCT GTT G
For fugu (*Fugu rubripes*)		
FuruPGI1-5'	*Pgi-1* (partial)	CAC GGA TGT AAA GAG CGT CTC CT
FuruPGI1-3'	*Pgi-1* (partial)	GCA GCA GTG GTA CAA AGC CAA

[1]Positions with mixed bases are designated by their IUB codes: R = A/G; Y = C/T; K = G/T; M = A/C; S = G/C; W = A/T.

Table S3. Gene-specific primers used for RT-PCR analysis of *Pgi* genes in this study.

Primers	Target gene (product size)	Sequence (5'→3')
For bichir (*Polypterus ornatipinnis*)		
PoorPGI-F	*Pgi* (491 bp)	TTC CAG CAG GGT GAC ATG GA
PoorPGI-R		ACA CCC CAC TGG TCA TAG CTG
For sturgeon (*Acipenser ruthenus*)		
AcruPGI-F	*Pgi* (344 bp)	ACA CCA AGG AAC ACG CAT GA
AcruPGI-R		AGC TGT TGA TGT CCC AGA TGA C
For amia (*Amia calva*)		
AmcaPGI-F	*Pgi* (422 bp)	GGG GCT CAC TGG ATG GAC AA
AmcaPGI-R		CCC TTC ATC AGG GCC TCA GT
For gar (*Lepisosteus osseus*)		
LeosPGI-F	*Pgi* (299 bp)	GGA GCT CAC TGG ATG GAC AA
LeosPGI-R		CCT TGA TGG ATG AGC TGG TAG A
For arowana (*Osteoglossum bicirrhosum*)		
OsbiPGI1-F	*Pgi-1* (266 bp)	ACG TGG TCA ATA TCG GTA TC
OsbiPGI1-R		AAC CAC AGA TGG ATC AGT AG
OsbiPGI2-F	*Pgi-2* (266 bp)	CGA TGT CGT CAA TAT TGG CAT T

OsbiPGI2-R		CAG CAG ATT TGT CCT TGG CG

For eel (*Anguilla anguilla*)

AgagPGI1-F	*Pgi-1* (285 bp)	TCA TCA AGG AAC ACG CAT GA
AgagPGI1-R		AGC GCT CCA AGA ATG AAA GG
AgagPGI2-F	*Pgi-2* (351 bp)	AAC CTG CAC CAC AAG ATC CT
AgagPGI2-R		AAC CTC AGT GGT GTC CTG AA

For zebrafish (*Danio rerio*)

DarePGI1-F	*Pgi-1* (243 bp)	GAG ATA ACC TGC ATC ATA AGA TC
DarePGI1-R		TCT TGT GCT CAT ACA TCG CA
DarePGI2-F	*Pgi-2* (179 bp)	CTG ATG AAG GGG AAA ACA ACA GAA
DarePGI2-R		TCA TAC ATG GCG ATC AGC ACA

For smelt (*Plecoglossus altivelis*)

PlalPGI1-F	*Pgi-1* (302 bp)	CAA GGC ACT CGT ATG ATT CC
PlalPGI1-R		TTG TGC TCG TAC ATT GCA AC
PlalPGI2-F	*Pgi-2* (357 bp)	ACT TAT CCA CCA AGG TAC TC
PlalPGI2-R		TGG TCA AAA CTG TTG ATC TC

For mullet (*Mugil cephalus*)

MucePGI1-F	*Pgi-1* (386 bp)	CCA AAG TAC TCG TCT GAT TCC
MucePGI1-R		TCT TCT TAG CGA GTT GCT TC
MucePGI2-F	*Pgi-2* (363 bp)	ATT GCT TTG CAC ATT GGC TTC

MucePGI2-R		AGA AGG CAC CAT TCG TGT TCC

For fugu (*Fugu rubripes*)

FuruPGI1-F	*Pgi-1* (311 bp)	GCT CAT CCA CCA AGG AAC TC
FuruPGI1-R		TGT GCT CGT ACA TGG CAA TC
FuruPGI2-F	*Pgi-2* (387 bp)	TGC GTG TAA ACT ACC ACA CT
FuruPGI2-R		TCT TGT GCT CAT ACA TCG CT

Table S4. Hierarchical likelihood ratio tests (hLRTs) among nested models of nucleotide substitution.

Models	Likelihood scores	Likelihood ratio tests[1]			
		F81 + Γ	HKY85 + Γ	TN93 + Γ	GTR + Γ
JC69 + Γ [3]	-16039	46.0* (3)	790.9* (4)	835.8* (5)	852.4* (8)
F81 + Γ [6]	-16016	-	744.9* (1)	789.8* (2)	806.4* (5)
HKY85 + Γ [7]	-15644	-	-	44.9* (1)	61.5* (4)
TN93 + Γ [8]	-15621	-	-	-	16.6* (3)
**GTR** + Γ [11]	-15613	-	-	-	-

*$P < 0.001$.

[1]The likelihood ratio test statistic (2ΔL) is approximated using the χ^2 distribution with degrees of freedom (in parentheses) equal to the difference in the number of parameters (in brackets) between the comparing pairs of nucleotide substitution models. The best-fitting model is indicated in **bold**.

Table S5. Inferred normalized amino acid frequencies (f_i) of the surface and interior portions of the common ancestral protein of PGI-1 and PGI-2 (node #5 in Figure 2C).

	Normalized frequency	
	Surface	Interior
Ala (A)	0.05556	0.09494
Arg (R)	0.05128	0.01582
Asn (N)	0.08120	0.04747
Asp (D)	0.06410	0.03165
Cys (C)	0.00000	0.00316
Gln (Q)	0.05983	0.02215
Glu (E)	0.14530	0.01266
Gly (G)	0.05556	0.08861
His (H)	0.03846	0.03165
Ile (I)	0.01709	0.09177
Leu (L)	0.02564	0.14241
Lys (K)	0.15812	0.01899
Met (M)	0.00855	0.05063
Phe (F)	0.02137	0.08228
Pro (P)	0.04274	0.03165
Ser (S)	0.04274	0.04747
Thr (T)	0.07265	0.06013
Trp (W)	0.02991	0.01582
Tyr (Y)	0.01709	0.03165
Val (V)	0.01282	0.07911

Supplementary Figure Legends

Fig. S1. Gene content around the *Pgi* locus (or loci) in the human, chicken, and zebrafish genomes according to the assembly versions of the human genome from October 2005 (NCBI 36), chicken genome from March 2004 (WASHUC 1), and zebrafish genome from March 2006 (Zv 6). The gray horizontal bars denote chromosomes with gene names showing their relative physical locations.

Fig. S2. Amino acid sequences of the PGI proteins analyzed in this study. The numbering of amino acid positions for these sequences was according to the published structures of the rabbit PGI protein [1]. The enzyme active sites are colored yellow; hydrophilic positively charged (basic) residues, blue; hydrophilic negatively charged (acidic) residues, red.

Fig. S3. Charge-changing (CC) substitutions in the evolution of fish PGIs mapped on the known phylogenetic tree [2] using a parsimony method with MacClade version 4.06 [3]. The numerals in bold type on the internal branch show the minimum–average–maximum numbers of CC substitutions estimated. Numbers shown in brackets refer to the amino acid position in the PGI protein [1] where the CC substitution event was estimated to have occurred. Squares and circles on the internal branches denote estimated unambiguous and ambiguous events, respectively. Shading indicates the consistency index (CI) value; a high CI indicates low homoplasy. The CC events with a high CI appeared near the tips of the tree rather than at deep internal nodes, which suggests parallel or recurring evolution of the electric charge.

Fig. S4. Patterns of sequence variation in *Pgi* nucleotide sequences from 20 vertebrate lineages. Transitional (Ts) and transversional (Tv) differences are plotted against pairwise evolutionary distances estimated by PAUP 4.0b10 [4] with the Tamura–Nei + I + Γ model [5], which was selected as the best-fit model of nucleotide substitution by Modeltest version 3.06 [6].

Fig. S5. Full-length gel images from the RT-PCR expression analysis of *Pgi* genes. The letters below each gel image indicate the tissues tested: M, muscle; L, liver; H, heart; Gi, gill; B, brain; Go, gonad; K, kidney; –, sterilized deionized water; GD, genomic DNA.

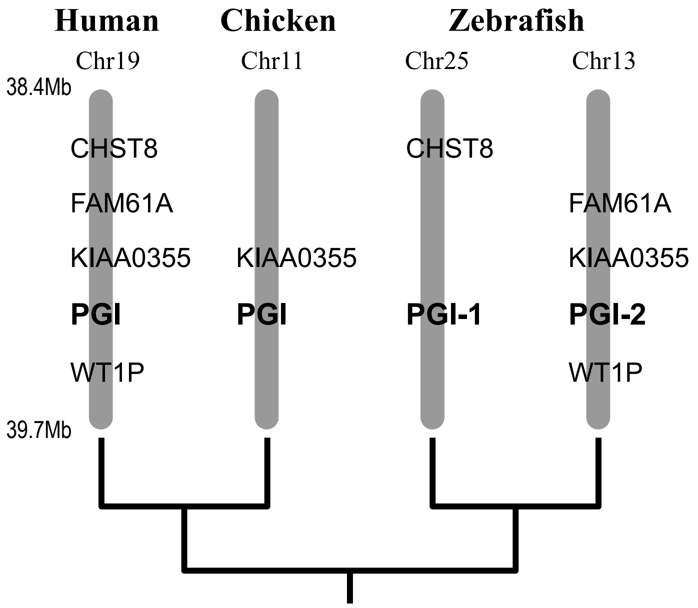

Sato and Nishida, Supplementary Fig. S1.

Position				1	2	3	4	5	6	7	8	9	10	11	12	13	14	15	16	17	18	19	20	21	22	23	24	25	26	27	28	29	30	31	32	33	34	35	36	37	38	39	40	41	42	43	44	45	46	47	48	49	50	51	52	53	54	55	56	57
Fugu-1	-	-	M	A	-	L	S	N	D	P	E	Y	R	K	L	Q	Q	W	Y	K	A	N	A	G	S	L	N	M	R	D	M	F	A	A	K	E	D	R	F	S	R	F	S	T	T	L	Q	T	E	E	G	D	I	L	L	D	F	S	K	N
Mullet-1	-	-	M	A	-	L	T	A	H	P	N	F	Q	K	L	Q	Q	W	F	K	A	Q	G	S	N	L	N	M	R	E	M	F	E	G	D	K	E	R	F	N	K	F	S	T	V	L	Q	T	D	D	G	E	I	L	L	D	F	S	K	N
Smelt-1	-	-	M	G	-	L	A	N	D	P	N	Y	Q	K	L	E	Q	W	Y	K	S	K	G	G	S	L	N	M	R	S	M	F	D	A	D	E	A	R	F	S	K	F	S	T	I	L	E	T	D	D	G	E	I	L	L	D	Y	S	K	N
Zebrafish-1	-	-	M	G	-	L	N	N	D	P	N	F	I	K	L	E	Q	W	Y	K	S	S	V	G	N	L	N	M	R	Q	M	F	E	A	D	A	D	R	F	N	K	F	S	L	Q	L	S	T	D	D	G	D	L	L	L	D	F	S	K	N
Eel-1	-	-	M	G	-	L	T	S	D	P	N	Y	K	K	L	E	Q	W	Y	R	S	N	A	G	N	L	N	M	R	Q	M	F	E	A	D	K	D	R	F	S	K	F	S	T	T	L	N	T	D	D	G	D	I	L	I	D	Y	S	K	N
Arowana-1	-	-	M	G	-	L	T	S	D	P	A	F	R	R	L	E	Q	W	Y	K	A	N	A	G	N	L	N	M	R	N	M	F	D	D	D	K	E	R	F	G	K	F	S	L	I	L	N	T	D	D	G	D	V	L	L	D	Y	S	K	N
Fugu-2	-	-	M	G	-	L	T	H	D	P	N	F	Q	K	L	Q	D	W	Y	T	A	H	A	L	N	I	N	L	R	H	M	F	E	A	D	K	E	R	F	N	K	L	S	L	T	L	K	T	D	D	G	D	I	L	L	D	Y	S	K	N
Mullet-2	-	-	M	G	-	L	T	Q	D	P	N	F	Q	K	L	Q	N	W	Y	T	A	H	A	L	N	L	N	L	R	H	M	F	E	A	D	K	E	R	F	N	K	L	S	L	T	L	K	T	E	D	G	D	I	L	L	D	Y	S	K	N
Smelt-2	-	-	M	G	-	L	T	S	D	P	N	F	Q	K	L	K	E	W	Y	K	A	H	A	L	S	L	N	L	R	Q	M	F	E	D	D	K	E	R	F	N	K	L	S	T	T	L	K	T	D	D	G	E	I	L	L	D	Y	S	K	N
Zebrafish-2	-	-	M	G	-	L	T	S	D	P	N	F	Q	N	L	E	K	W	Y	K	S	N	A	A	N	L	N	M	R	Q	M	F	E	E	D	K	T	R	F	Q	K	F	S	L	T	L	K	T	D	V	G	D	I	L	L	D	Y	S	K	N
Eel-2	-	-	M	G	-	L	T	S	D	P	N	Y	Q	K	L	E	Q	W	F	K	T	H	A	G	S	L	S	M	R	Q	M	F	D	A	D	K	D	R	F	S	K	F	S	T	T	L	K	T	D	D	G	D	I	L	L	D	Y	S	K	N
Arowana-2	-	-	M	G	-	L	T	S	D	P	S	F	Q	K	L	D	Q	W	S	K	S	H	A	R	T	L	N	M	R	H	M	F	D	S	D	K	D	R	F	G	K	F	S	T	T	L	K	T	E	D	G	D	I	L	L	D	Y	S	K	N
Sturgeon	M	A	S	G	-	L	T	S	D	P	N	F	Q	K	L	Q	Q	W	H	K	D	K	A	A	S	L	K	M	R	Q	M	F	Q	A	D	K	D	R	F	N	K	L	S	T	T	L	K	T	D	D	G	D	I	L	I	D	Y	S	K	N
Gar	M	A	P	G	-	L	T	S	D	P	S	F	Q	K	L	E	Q	W	Y	R	A	N	A	G	S	L	N	M	R	Q	M	F	E	A	D	Q	G	R	F	G	K	F	S	T	T	L	N	T	D	D	G	D	I	L	V	D	F	S	K	N
Amia	M	A	P	G	-	L	T	S	D	P	S	Y	K	K	L	E	K	W	Y	K	S	N	A	G	N	L	N	M	R	Q	M	F	D	A	D	K	Q	R	F	S	K	F	S	T	T	L	K	T	D	E	G	D	L	L	I	D	Y	S	K	N
Bichir	M	A	S	G	G	L	T	S	D	P	N	F	Q	K	L	E	Q	W	Y	K	A	N	G	A	A	L	N	L	R	Q	L	F	D	S	D	H	D	R	F	K	K	F	S	T	T	L	Q	T	S	D	G	D	I	L	I	D	Y	S	K	N
Mouse	-	-	M	A	A	L	T	R	N	P	Q	F	Q	K	L	L	E	W	H	R	A	N	S	A	N	L	K	L	R	E	L	F	E	A	D	P	E	R	F	N	N	F	S	L	N	L	N	T	N	H	G	H	I	L	V	D	Y	S	K	N
Rat	-	-	M	A	A	L	T	R	N	P	E	F	Q	K	L	L	E	W	H	R	A	N	S	A	N	L	K	L	R	E	L	F	E	A	D	P	E	R	F	N	H	F	S	L	N	L	N	T	N	H	G	H	I	L	L	D	Y	S	K	N
Hamster	-	-	M	T	S	L	T	Q	N	R	Y	F	Q	K	L	Q	D	W	H	R	D	N	S	A	D	I	N	L	R	S	L	F	D	A	D	P	E	R	F	N	N	F	S	L	N	L	N	T	T	H	G	H	I	L	V	D	Y	S	K	N
Pig	-	-	M	A	A	L	T	Q	N	P	Q	F	K	K	L	Q	T	W	Y	H	E	H	R	S	D	L	N	L	R	R	L	F	E	G	D	K	D	R	F	N	H	F	S	L	N	L	N	T	N	H	G	R	I	L	L	D	Y	S	K	N
Rabbit	-	-	M	A	A	L	T	R	N	P	Q	F	Q	K	L	Q	Q	W	H	R	E	H	G	S	E	L	N	L	R	H	L	F	D	T	D	K	E	R	F	N	H	F	S	L	T	L	N	T	N	H	G	H	I	L	L	D	Y	S	K	N
Human	-	-	M	A	A	L	T	R	D	P	Q	F	Q	K	L	Q	Q	W	Y	R	E	H	R	S	E	L	N	L	R	R	L	F	D	A	N	K	D	R	F	N	H	F	S	L	T	L	N	T	N	H	G	H	I	L	V	D	Y	S	K	N
Snake	-	-	M	A	-	L	S	A	D	P	C	F	Q	R	L	A	E	W	Q	R	A	H	T	G	Q	L	V	L	R	K	L	F	Q	D	D	P	E	R	F	H	K	L	S	V	T	L	K	A	E	H	G	D	I	L	V	D	Y	S	K	N
Chicken	-	-	M	P	-	L	S	A	D	P	H	F	K	K	L	L	E	W	H	K	A	N	A	S	K	L	V	L	R	Q	L	F	E	A	D	K	D	R	F	H	K	F	S	L	T	L	N	T	D	H	G	D	I	L	L	D	Y	S	K	N
Toad	-	-	M	A	-	L	T	S	D	P	V	Y	Q	K	L	A	K	F	Y	E	A	N	H	G	C	L	N	L	R	K	L	F	E	A	D	K	E	R	F	N	K	F	S	K	T	L	N	T	G	E	G	D	I	L	V	D	Y	S	K	N
Hagfish	-	-	M	A	T	L	T	A	D	P	A	W	R	K	L	S	A	W	Y	S	A	H	G	A	S	L	N	L	R	K	M	F	D	A	D	K	E	R	F	S	K	F	S	R	V	L	K	T	D	D	G	D	I	L	L	D	F	S	K	N

Supplementary Fig. S2 (to be continued).

Position	58	59	60	61	62	63	64	65	66	67	68	69	70	71	72	73	74	75	76	77	78	79	80	81	82	83	84	85	86	87
Fugu-1	L	I	N	Q	E	V	L	D	M	L	L	L	L	A	R	S	R	G	V	E	E	A	R	Q	K	M	F	S	G	E
Mullet-1	L	I	N	Q	D	V	L	A	Q	L	L	A	M	A	K	S	R	G	V	E	E	A	R	D	R	M	F	S	G	E
Smelt-1	L	I	N	D	E	V	M	Q	M	L	L	A	M	A	K	S	R	G	V	E	E	A	R	E	R	M	F	S	G	E
Zebrafish-1	L	I	N	E	D	V	M	S	L	L	F	D	M	A	R	S	V	G	V	E	A	A	R	E	Q	M	I	A	G	E
Eel-1	L	I	N	E	E	V	M	K	M	L	I	A	M	A	K	S	R	G	V	E	A	A	R	E	K	M	F	T	G	E
Arowana-1	L	I	D	E	D	V	L	G	M	L	L	D	L	A	K	S	R	G	V	E	A	A	R	E	R	M	F	T	G	E
Fugu-2	L	I	T	D	D	V	M	K	M	L	V	D	L	A	K	S	R	G	I	E	A	A	R	E	K	M	F	T	G	E
Mullet-2	L	I	T	D	E	V	V	K	M	L	V	D	L	A	K	S	R	G	I	E	A	A	R	E	R	M	F	S	G	E
Smelt-2	L	L	T	E	D	V	V	K	M	L	V	E	L	A	K	S	R	G	V	D	A	A	R	E	K	M	F	S	G	E
Zebrafish-2	L	I	N	E	E	V	M	K	M	L	V	E	L	A	K	S	R	G	V	E	A	S	R	D	K	M	F	S	G	E
Eel-2	L	I	N	E	E	V	M	H	M	L	V	D	L	A	K	S	R	G	V	E	A	A	R	E	R	M	F	T	G	E
Arowana-2	L	I	T	E	E	V	M	K	M	L	V	D	L	A	K	S	R	G	V	E	A	G	R	E	R	M	F	T	G	E
Sturgeon	L	I	T	E	E	V	M	K	M	L	V	D	L	A	K	S	R	G	V	E	Q	A	R	E	K	M	F	T	G	E
Gar	L	I	N	E	E	V	M	K	M	L	F	D	L	A	R	S	R	G	V	E	E	A	R	E	K	M	F	T	G	E
Amia	L	I	T	E	E	V	L	K	M	L	Y	E	M	A	K	S	R	G	V	L	E	A	R	E	R	M	F	K	G	E
Bichir	L	I	T	E	E	V	M	K	M	L	V	E	L	A	K	S	R	G	V	E	K	A	R	E	R	M	F	T	G	E
Mouse	L	V	N	K	E	V	M	Q	M	L	V	E	L	A	K	S	R	G	V	E	A	A	R	D	N	M	F	S	G	S
Rat	L	V	N	K	E	V	L	H	M	L	V	D	L	A	K	S	R	G	V	E	A	A	R	D	N	M	F	S	G	L
Hamster	L	V	N	K	E	V	M	Q	M	L	V	D	L	A	R	S	R	G	V	E	T	M	R	D	N	M	F	S	G	V
Pig	L	V	T	E	A	V	M	Q	M	L	V	D	L	A	K	S	R	G	V	E	A	A	R	E	R	M	F	N	G	E
Rabbit	L	V	T	E	E	V	M	H	M	L	L	D	L	A	K	S	R	G	V	E	A	A	R	E	S	M	F	N	G	E
Human	L	V	T	E	D	V	M	R	M	L	V	D	L	A	K	S	R	G	V	E	A	A	R	E	R	M	F	N	G	E
Snake	L	V	T	E	E	V	M	K	M	L	V	G	L	A	K	S	R	G	V	E	K	A	R	Q	Q	M	F	T	G	E
Chicken	L	V	T	E	E	V	M	K	M	L	I	E	L	A	K	S	R	G	V	E	S	A	R	E	R	M	F	S	G	E
Toad	L	I	T	E	E	V	I	K	L	L	I	E	L	A	R	S	R	G	V	E	A	A	R	K	R	M	F	S	A	E
Hagfish	L	I	T	Q	E	T	F	D	L	L	L	N	L	A	R	S	R	S	V	E	A	A	R	T	R	M	F	S	G	E

Position	88	89	90	91	92	93	94	95	96	97	98	99	100	101	102	103	104	105	106	107	108	109	110	111	112	113	114	115	116	117
Fugu-1	K	I	N	F	T	E	G	R	A	V	L	H	V	A	L	R	N	R	S	N	E	P	I	C	V	D	G	T	D	V
Mullet-1	K	I	N	F	T	E	G	R	A	V	L	H	V	A	L	R	N	R	S	N	S	P	I	L	V	D	G	K	D	V
Smelt-1	K	I	N	F	T	E	G	R	A	V	L	H	I	A	L	R	N	R	S	N	T	P	V	L	V	D	G	S	D	V
Zebrafish-1	K	I	N	F	T	E	G	R	A	V	L	H	T	A	L	W	N	R	S	N	T	P	I	M	V	D	G	K	D	V
Eel-1	K	I	N	F	T	E	G	R	A	V	L	H	T	A	L	R	N	R	S	N	T	P	I	H	V	G	G	T	D	V
Arowana-1	K	I	N	F	T	E	G	R	A	V	L	H	I	A	L	R	N	R	S	N	T	P	I	C	V	G	G	K	D	V
Fugu-2	K	I	N	F	T	E	G	R	A	V	L	H	V	A	L	R	N	R	S	N	K	P	I	I	V	D	G	K	D	V
Mullet-2	K	I	N	F	T	E	G	R	A	V	L	H	V	A	L	R	N	R	S	N	T	P	I	V	V	G	G	K	D	V
Smelt-2	K	I	N	F	T	E	G	R	A	V	L	H	V	A	L	R	N	R	S	N	T	P	I	N	V	E	G	K	D	V
Zebrafish-2	K	I	N	F	T	E	G	R	A	V	L	H	V	A	L	R	N	R	S	N	T	P	I	N	V	D	G	K	D	V
Eel-2	K	I	N	F	T	E	G	R	A	V	L	H	V	A	L	R	N	R	S	N	T	P	I	M	V	D	G	Q	D	V
Arowana-2	K	I	N	F	T	E	G	R	A	V	L	H	V	A	L	R	N	R	S	N	T	P	I	V	V	D	G	K	D	V
Sturgeon	Q	I	N	F	T	E	G	R	A	V	L	H	I	A	L	R	N	R	S	N	S	P	I	N	V	A	G	K	D	V
Gar	K	I	N	F	T	E	N	R	A	V	L	H	I	A	L	R	N	R	S	N	S	P	I	H	V	G	G	K	D	V
Amia	K	I	N	F	T	E	G	R	A	V	L	H	I	A	L	R	N	R	S	N	S	P	I	E	V	D	G	K	D	V
Bichir	K	I	N	F	T	E	N	R	A	V	L	H	I	A	L	R	N	R	S	N	T	P	I	L	V	N	G	K	D	V
Mouse	K	I	N	Y	T	E	D	R	A	V	L	H	V	A	L	R	N	R	S	N	T	P	I	K	V	D	G	K	D	V
Rat	K	I	N	S	T	E	D	R	A	V	L	H	V	A	L	R	N	R	S	N	R	S	I	M	M	D	G	K	D	V
Hamster	K	I	N	Y	T	E	D	R	A	V	L	H	V	A	L	R	N	R	S	N	S	P	I	V	V	D	S	R	D	V
Pig	K	I	N	F	T	E	D	R	A	V	L	H	V	A	L	R	N	R	S	N	T	P	I	L	V	D	G	K	D	V
Rabbit	K	I	N	S	T	E	D	R	A	V	L	H	V	A	L	R	N	R	S	N	T	P	I	V	V	D	G	K	D	V
Human	K	I	N	Y	T	E	G	R	A	V	L	H	V	A	L	R	N	R	S	N	T	P	I	L	V	D	G	K	D	V
Snake	K	I	N	F	T	E	N	R	A	V	L	H	I	A	L	R	N	R	S	N	T	P	I	L	V	E	G	K	D	V
Chicken	K	I	N	F	T	E	N	R	A	V	L	H	I	A	L	R	N	R	S	N	T	P	I	L	V	D	G	K	D	V
Toad	K	I	N	F	T	E	N	R	A	V	L	H	I	A	L	R	N	R	S	N	A	P	I	Q	V	D	G	K	D	M
Hagfish	K	I	N	F	T	E	N	R	A	V	F	H	V	A	L	R	N	R	S	G	H	P	M	I	V	D	G	Q	D	V

Supplementary Fig. S2 (continued).

Position	118	119	120	121	122	123	124	125	126	127	128	129	130	131	132	133	134	135	136	137	138	139	140	141	142	143	144	145	146	147	148	149	150	151	152	153	154	155	156	157	158	159	160	161	162	163	164	165	166	167	168	169	170	171	172	173	174	175	176	177
Fugu-1	M	P	E	V	N	R	V	L	E	K	M	K	T	F	C	H	R	V	R	S	G	E	W	K	G	F	S	G	K	S	I	T	D	V	V	N	I	G	I	G	G	S	D	L	G	P	L	M	V	T	E	A	L	K	P	Y	S	A	G	G
Mullet-1	M	P	E	V	N	R	V	L	D	K	M	K	V	F	C	Q	K	V	R	S	G	D	W	K	G	F	S	G	K	S	I	T	D	V	V	N	I	G	I	G	G	S	D	L	G	P	L	M	V	T	E	A	L	K	P	Y	S	T	G	G
Smelt-1	M	T	D	V	N	R	V	L	D	K	M	K	A	F	C	H	K	V	R	S	G	D	W	K	G	F	S	G	K	A	I	T	D	V	V	N	I	G	I	G	G	S	D	L	G	P	L	M	V	T	E	A	L	K	P	Y	S	E	G	G
Zebrafish-1	M	P	E	V	N	R	V	L	E	Q	M	K	S	F	C	H	K	V	R	S	G	A	W	K	G	F	S	G	K	S	I	T	D	V	V	N	I	G	I	G	G	S	D	L	G	P	L	M	V	T	E	A	L	K	P	Y	S	K	G	G
Eel-1	M	P	E	V	N	R	V	L	E	K	M	K	N	F	C	H	K	V	R	S	G	E	W	K	G	F	T	G	Q	A	I	T	D	V	V	N	V	G	I	G	G	S	D	L	G	P	L	M	V	T	E	A	L	K	P	Y	S	K	G	G
Arowana-1	M	P	D	V	N	R	V	L	E	K	M	K	G	F	C	H	K	V	R	S	G	E	W	K	G	Y	T	G	K	A	I	T	D	V	V	N	I	G	I	G	G	S	D	L	G	P	L	M	V	T	E	A	L	K	P	Y	S	T	G	G
Fugu-2	M	P	G	V	N	N	V	L	E	K	M	K	V	F	S	H	K	V	R	S	G	E	W	K	G	Y	T	G	K	A	I	T	D	V	V	N	V	G	I	G	G	S	D	L	G	P	L	M	V	T	E	A	L	K	P	Y	S	K	G	G
Mullet-2	M	P	E	V	N	K	V	L	E	K	M	K	G	F	C	H	R	V	R	S	G	E	W	K	G	Y	T	G	K	A	I	T	D	V	V	N	V	G	I	G	G	S	D	L	G	P	L	M	V	T	E	A	L	K	P	Y	S	K	G	G
Smelt-2	M	P	E	V	N	K	V	L	E	K	M	K	G	F	C	H	K	V	R	S	G	D	W	K	G	Y	T	G	K	A	I	T	D	V	V	N	V	G	I	G	G	S	D	L	G	P	L	M	V	T	E	A	L	K	P	Y	S	K	D	G
Zebrafish-2	M	P	E	V	N	K	V	L	E	K	M	K	G	F	C	H	K	V	R	S	G	E	W	K	G	F	T	G	K	S	I	T	D	V	V	N	V	G	I	G	G	S	D	L	G	P	L	M	V	T	E	A	L	K	P	Y	S	K	G	G
Eel-2	T	P	E	V	N	K	V	L	E	K	M	K	G	F	C	H	K	V	R	S	G	E	W	K	G	Y	T	G	K	S	I	T	D	V	V	N	V	G	I	G	G	S	D	L	G	P	L	M	V	T	E	A	L	K	P	Y	S	K	G	G
Arowana-2	M	P	E	V	N	K	V	L	E	K	M	K	G	F	C	H	K	V	R	S	G	E	W	K	G	Y	T	G	K	S	I	T	D	V	V	N	I	G	I	G	G	S	D	L	G	P	L	M	V	T	E	A	L	K	P	Y	S	K	G	G
Sturgeon	M	P	E	V	N	K	V	L	E	K	M	K	N	F	C	Q	K	V	R	S	G	D	W	K	G	Y	T	G	K	S	I	T	D	V	V	N	I	G	I	G	G	S	D	L	G	P	L	M	V	T	E	A	L	K	P	Y	S	K	G	G
Gar	M	P	E	V	N	S	V	L	E	K	M	K	A	F	C	Q	K	V	R	S	G	E	W	K	G	Y	T	G	K	A	I	T	D	I	V	N	I	G	I	G	G	S	D	L	G	P	L	M	V	T	E	A	L	K	P	Y	S	K	G	G
Amia	M	P	D	V	N	N	V	L	E	K	M	K	G	F	C	Q	K	V	R	S	G	D	W	K	G	Y	S	G	K	P	I	T	D	V	I	N	I	G	I	G	G	S	D	L	G	P	L	M	V	T	E	A	L	K	P	Y	S	K	G	G
Bichir	M	P	E	V	N	N	V	L	E	K	M	K	K	F	C	Q	K	V	R	S	G	D	W	K	G	Y	T	G	K	A	I	T	D	V	V	N	I	G	I	G	G	S	D	L	G	P	L	M	V	T	E	A	L	K	P	Y	S	K	G	G
Mouse	M	P	E	V	N	R	V	L	D	K	M	K	S	F	C	Q	R	V	R	S	G	D	W	K	G	Y	T	G	K	S	I	T	D	I	I	N	I	G	I	G	G	S	D	L	G	P	L	M	V	T	E	A	L	K	P	Y	S	K	G	G
Rat	M	P	E	V	N	K	V	L	D	K	M	K	S	F	C	Q	R	V	R	S	G	D	W	K	G	Y	T	G	K	A	I	T	D	I	I	N	I	G	I	G	G	S	D	L	G	P	L	M	V	T	E	A	L	K	P	Y	S	K	G	G
Hamster	M	P	E	V	N	R	V	L	E	K	M	R	S	F	C	Q	R	V	R	S	G	E	W	K	G	Y	S	G	K	P	I	T	D	I	V	N	I	G	I	G	G	S	D	L	G	P	L	M	V	T	E	A	L	K	P	Y	A	S	G	G
Pig	M	P	E	V	N	R	V	L	E	K	M	K	S	F	C	K	R	V	R	S	G	E	W	K	G	Y	S	G	K	S	I	T	D	V	I	N	I	G	I	G	G	S	D	L	G	P	L	M	V	T	E	A	L	K	P	Y	S	A	E	G
Rabbit	M	P	E	V	N	K	V	L	D	K	M	K	A	F	C	Q	R	V	R	S	G	D	W	K	G	Y	T	G	K	T	I	T	D	V	I	N	I	G	I	G	G	S	D	L	G	P	L	M	V	T	E	A	L	K	P	Y	S	S	G	G
Human	M	P	E	V	N	K	V	L	D	K	M	K	S	F	C	Q	R	V	R	S	G	D	W	K	G	Y	T	G	K	T	I	T	D	V	I	N	I	G	I	G	G	S	D	L	G	P	L	M	V	T	E	A	L	K	P	Y	S	S	G	G
Snake	V	P	E	V	N	Q	V	L	E	K	M	K	N	F	C	Q	K	V	R	S	G	D	W	K	G	Y	T	G	K	A	I	T	D	V	V	N	I	G	I	G	G	S	D	L	G	P	L	M	V	T	E	A	L	K	P	Y	S	K	G	G
Chicken	V	P	E	V	N	K	V	L	D	K	M	K	H	F	C	Q	K	V	R	S	G	E	W	K	G	Y	T	G	K	A	I	T	D	V	V	N	I	G	I	G	G	S	D	L	G	P	L	M	V	T	E	A	L	K	P	Y	S	K	G	G
Toad	M	P	E	V	N	A	V	L	G	K	M	K	T	F	C	Q	K	V	R	S	G	D	W	K	G	Y	S	G	K	A	I	T	D	V	V	N	V	G	I	G	G	S	D	L	G	P	L	M	V	T	E	A	L	K	P	Y	S	K	G	G
Hagfish	M	P	A	V	N	A	V	L	E	K	M	K	K	F	C	H	R	V	R	S	G	E	W	K	G	Y	T	G	K	A	I	T	D	V	V	N	I	G	I	G	G	S	D	L	G	P	L	M	V	C	E	S	L	K	P	Y	S	K	G	G

Supplementary Fig. S2 (continued).

Position	178	179	180	181	182	183	184	185	186	187	188	189	190	191	192	193	194	195	196	197	198	199	200	201	202	203	204	205	206	207
Fugu-1	P	N	V	W	F	V	S	N	I	D	G	T	H	M	A	K	T	L	A	K	L	D	A	E	T	T	L	F	I	I
Mullet-1	P	K	V	W	F	V	S	N	I	D	G	T	H	M	A	K	T	L	A	Q	L	D	P	E	T	T	L	F	I	I
Smelt-1	P	N	V	W	F	V	S	N	I	D	G	T	H	M	A	K	T	L	A	K	L	N	A	E	T	T	L	F	I	I
Zebrafish-1	P	N	V	W	F	V	S	N	I	D	G	T	H	M	A	K	T	L	A	Q	L	N	A	E	T	T	L	F	I	I
Eel-1	P	R	V	W	F	V	S	N	I	D	G	T	H	M	A	K	T	L	A	D	L	N	P	E	S	T	L	F	I	V
Arowana-1	P	R	A	W	F	V	S	N	I	D	G	T	H	I	A	K	T	L	A	G	L	S	P	E	T	T	L	F	I	V
Fugu-2	P	H	V	W	F	V	S	N	I	D	G	T	H	I	A	K	T	L	E	Q	L	N	A	E	T	T	L	F	I	I
Mullet-2	P	R	V	W	F	V	S	N	I	D	G	T	H	I	A	K	T	L	A	Q	L	N	A	E	T	T	L	F	I	I
Smelt-2	P	R	V	W	F	V	S	N	I	D	G	T	H	I	A	K	T	L	A	Q	L	N	A	E	T	T	L	F	I	V
Zebrafish-2	P	R	V	W	F	V	S	N	I	D	G	T	H	I	A	K	T	L	A	E	L	N	A	E	T	T	L	F	I	I
Eel-2	P	R	V	W	F	V	S	N	I	D	G	T	H	I	A	K	T	L	A	E	L	N	A	E	T	T	L	F	I	V
Arowana-2	P	R	V	W	F	V	S	N	I	D	G	T	H	I	A	K	T	L	A	E	L	N	A	E	T	T	L	F	I	I
Sturgeon	P	K	A	W	F	V	S	N	I	D	G	T	H	M	A	K	T	L	A	E	L	N	P	E	T	T	L	F	I	I
Gar	P	R	A	W	F	V	S	N	I	D	G	T	H	M	A	K	T	L	A	E	L	N	P	E	T	T	L	F	I	I
Amia	P	R	V	W	F	V	S	N	I	D	G	T	H	M	A	K	T	L	A	E	L	D	P	E	T	S	L	F	I	I
Bichir	P	K	V	W	F	V	S	N	I	D	G	T	H	M	A	K	T	L	A	E	L	N	P	E	T	T	L	F	I	I
Mouse	P	R	V	W	F	V	S	N	I	D	G	T	H	I	A	K	T	L	A	S	L	S	P	E	T	S	L	F	I	I
Rat	P	R	V	W	F	V	S	N	I	D	G	T	H	I	A	K	T	L	A	N	L	N	P	E	S	S	L	F	I	I
Hamster	P	R	I	W	F	V	S	N	I	D	G	T	H	I	A	K	T	L	A	N	L	T	P	E	S	S	L	F	I	V
Pig	P	R	V	W	F	V	S	N	I	D	G	T	H	I	A	K	T	L	A	T	L	N	P	E	S	S	L	F	I	I
Rabbit	P	R	V	W	F	V	S	N	I	D	G	T	H	I	A	K	T	L	A	C	L	N	P	E	S	S	L	F	I	I
Human	P	R	V	W	Y	V	S	N	I	D	G	T	H	I	A	K	T	L	A	Q	L	N	P	E	S	S	L	F	I	I
Snake	P	R	V	W	F	V	S	N	I	D	G	T	H	M	A	K	T	L	A	K	L	N	P	E	T	V	L	F	I	I
Chicken	P	R	V	W	F	V	S	N	I	D	G	T	H	I	A	K	T	L	A	E	L	H	P	E	T	T	L	F	I	I
Toad	P	R	V	W	F	V	S	N	I	D	G	T	H	M	A	K	T	L	A	E	L	N	P	E	T	T	L	F	I	V
Hagfish	P	H	A	W	F	V	S	N	I	D	G	T	H	L	A	K	T	L	T	K	L	N	P	E	S	T	L	F	I	I

Position	208	209	210	211	212	213	214	215	216	217	218	219	220	221	222	223	224	225	226	227	228	229	230	231	232	233	234	235	236	237
Fugu-1	A	S	K	T	F	T	T	Q	E	T	I	T	N	A	E	S	A	K	D	W	F	L	Q	T	A	S	D	K	S	A
Mullet-1	A	S	K	T	F	T	T	Q	E	T	I	T	N	A	E	S	A	R	D	W	F	L	Q	T	A	G	D	K	S	A
Smelt-1	A	S	K	T	F	T	T	Q	E	T	I	T	N	A	E	T	A	R	E	W	L	L	N	T	A	N	D	K	S	A
Zebrafish-1	A	S	K	T	F	T	T	Q	E	T	I	T	N	A	E	T	A	R	E	W	F	L	Q	A	A	K	D	K	S	A
Eel-1	A	S	K	T	F	T	T	Q	E	T	I	T	N	A	E	T	A	K	E	W	L	L	Q	N	F	Q	D	K	S	S
Arowana-1	A	S	K	T	F	T	T	Q	E	T	I	T	N	A	E	S	A	K	E	W	L	L	Q	A	A	T	D	P	S	V
Fugu-2	A	S	K	T	F	T	T	Q	E	T	I	T	N	A	E	S	A	K	A	W	F	L	Q	H	A	K	D	S	A	A
Mullet-2	A	S	K	T	F	T	T	Q	E	T	I	T	N	A	E	S	A	K	A	W	F	L	E	H	A	K	D	K	A	A
Smelt-2	A	S	K	T	F	T	T	Q	E	T	I	T	N	A	E	S	A	K	E	W	F	L	S	A	A	K	D	K	S	A
Zebrafish-2	A	S	K	T	F	T	T	Q	E	T	I	T	N	A	E	S	A	K	E	W	F	L	Q	A	A	K	D	A	S	A
Eel-2	A	S	K	T	F	T	T	Q	E	T	I	T	N	A	E	S	A	K	E	W	F	L	A	A	A	K	D	P	A	A
Arowana-2	A	S	K	T	F	T	T	Q	E	T	I	T	N	A	E	S	A	K	E	W	F	L	N	A	A	K	D	K	S	A
Sturgeon	A	S	K	T	F	T	T	Q	E	T	I	T	N	A	E	S	A	K	E	W	F	L	Q	S	A	G	D	K	S	A
Gar	A	S	K	T	F	T	T	Q	E	T	I	T	N	A	E	S	A	K	E	W	L	L	Q	K	A	G	D	K	S	A
Amia	A	S	K	T	F	T	T	Q	E	T	I	T	N	A	E	S	A	K	E	W	F	L	Q	K	A	G	D	A	S	A
Bichir	A	S	K	T	F	T	T	Q	E	T	I	T	N	A	E	S	A	K	E	W	F	L	Q	A	A	V	D	K	S	A
Mouse	A	S	K	T	F	T	T	Q	E	T	I	T	N	A	E	T	A	K	E	W	F	L	E	A	A	K	D	P	S	A
Rat	A	S	K	T	F	T	T	Q	E	T	I	T	N	A	E	T	A	K	E	W	F	L	Q	A	A	K	D	P	S	A
Hamster	A	S	K	T	F	T	T	Q	E	T	I	T	N	A	E	T	A	K	E	W	F	L	G	A	S	R	D	P	S	T
Pig	A	S	K	T	F	T	T	Q	E	T	I	T	N	A	E	T	A	K	E	W	F	L	Q	S	A	K	D	P	S	A
Rabbit	A	S	K	T	F	T	T	Q	E	T	I	T	N	A	E	T	A	K	D	W	F	L	L	S	A	K	D	P	S	T
Human	A	S	K	T	F	T	T	Q	E	T	I	T	N	A	E	T	A	K	E	W	F	L	Q	A	A	K	D	P	S	A
Snake	A	S	K	T	F	T	T	Q	E	T	I	T	N	A	E	T	A	K	E	W	F	L	Q	A	A	K	D	P	S	A
Chicken	A	S	K	T	F	T	T	Q	E	T	I	T	N	A	V	T	A	K	E	W	F	L	H	A	A	K	D	P	S	A
Toad	A	S	K	T	F	T	T	Q	E	T	I	T	N	A	E	T	A	K	E	W	F	L	K	A	A	K	D	P	S	A
Hagfish	A	S	K	T	F	T	T	Q	E	T	I	T	N	A	N	S	A	K	E	W	F	L	E	T	A	K	D	P	A	T

Supplementary Fig. S2 (continued).

Position	238	239	240	241	242	243	244	245	246	247	248	249	250	251	252	253	254	255	256	257	258	259	260	261	262	263	264	265	266	267
Fugu-1	V	A	K	H	F	V	A	L	S	T	N	S	A	K	V	R	D	F	G	I	D	T	E	N	M	F	E	F	W	D
Mullet-1	V	A	K	H	F	V	A	L	S	T	N	A	P	K	V	K	D	F	G	I	D	T	E	N	M	F	E	F	W	D
Smelt-1	V	A	K	H	F	V	A	L	S	T	N	A	P	K	V	K	D	F	G	I	D	T	N	N	M	F	E	F	W	D
Zebrafish-1	V	A	K	H	F	V	A	L	S	T	N	A	P	K	V	K	E	F	G	I	D	T	N	N	M	F	E	F	W	D
Eel-1	V	A	K	H	F	V	A	L	S	T	N	A	P	K	V	K	D	F	G	I	N	P	D	N	M	F	E	F	W	D
Arowana-1	V	A	K	H	F	V	A	L	S	T	N	A	P	K	V	K	E	F	G	I	D	T	E	N	M	F	E	F	W	D
Fugu-2	V	A	K	H	F	V	A	L	S	T	N	G	P	K	V	K	D	F	G	I	D	T	E	N	M	F	E	F	W	D
Mullet-2	V	A	K	H	F	V	A	L	S	T	N	G	P	K	V	K	D	F	G	I	D	T	E	N	M	F	E	F	W	D
Smelt-2	V	A	K	H	F	V	A	L	S	T	N	A	P	K	V	K	D	F	G	I	D	T	E	N	M	F	E	F	W	D
Zebrafish-2	V	A	K	H	F	V	A	L	S	T	N	G	P	K	V	K	D	F	G	I	D	P	E	N	M	F	E	F	W	D
Eel-2	V	S	K	H	F	V	A	L	S	T	N	G	P	K	V	K	D	F	G	I	D	P	E	N	M	F	G	F	W	D
Arowana-2	V	A	K	H	F	A	A	L	S	T	N	A	P	K	V	K	D	F	G	I	D	T	E	N	M	F	E	F	W	D
Sturgeon	V	A	K	H	F	V	A	L	S	T	N	A	P	K	V	K	D	F	G	I	D	T	N	N	M	F	E	F	W	D
Gar	V	A	K	H	F	V	A	L	S	T	N	A	P	K	V	K	D	F	G	I	D	T	N	N	M	F	E	F	W	D
Amia	V	A	K	H	F	V	A	L	S	T	N	A	P	K	V	K	E	F	G	I	D	T	N	N	M	F	E	F	W	D
Bichir	V	A	K	H	F	V	A	L	S	T	N	A	P	K	V	K	D	F	G	I	D	T	N	N	M	F	E	F	W	D
Mouse	V	A	K	H	F	V	A	L	S	T	N	T	A	K	V	K	E	F	G	I	D	P	Q	N	M	F	E	F	W	D
Rat	V	A	K	H	F	V	A	L	S	T	N	T	D	K	V	K	E	F	G	I	D	P	K	N	M	F	E	F	W	D
Hamster	V	A	K	H	F	I	A	L	S	T	N	T	S	K	V	K	E	F	G	I	D	P	Q	N	M	F	E	F	W	D
Pig	V	A	K	H	F	V	A	L	S	T	N	T	T	K	V	K	E	F	G	I	D	P	Q	N	M	F	E	F	W	D
Rabbit	V	A	K	H	F	V	A	L	S	T	N	T	A	K	V	K	E	F	G	I	D	P	Q	N	M	F	E	F	W	D
Human	V	A	K	H	F	V	A	L	S	T	N	T	T	K	V	K	E	F	G	I	D	P	Q	N	M	F	E	F	W	D
Snake	V	A	K	H	F	A	A	L	S	T	N	G	P	K	V	K	D	F	G	I	D	T	A	N	M	F	E	F	W	D
Chicken	V	A	K	H	F	V	A	L	S	T	N	G	P	K	V	K	E	F	G	I	D	T	E	N	M	F	E	F	W	D
Toad	V	A	K	H	F	V	A	L	S	T	N	A	P	K	V	K	D	F	G	I	D	T	A	N	M	F	E	F	W	D
Hagfish	V	A	K	H	F	V	A	L	S	T	N	G	P	K	V	A	E	F	G	I	D	T	N	N	M	F	E	F	W	D

Position	268	269	270	271	272	273	274	275	276	277	278	279	280	281	282	283	284	285	286	287	288	289	290	291	292	293	294	295	296	297
Fugu-1	W	V	G	G	R	Y	S	L	W	S	A	I	G	L	S	I	A	L	H	V	G	F	E	H	F	E	Q	L	L	S
Mullet-1	W	V	G	G	R	Y	S	L	W	S	A	I	G	L	S	I	A	L	H	I	G	F	E	N	F	E	Q	L	L	S
Smelt-1	W	V	G	G	R	Y	S	L	W	S	A	I	G	L	S	I	A	L	H	I	G	F	D	N	F	E	Q	L	L	S
Zebrafish-1	W	V	G	G	R	Y	S	L	W	S	A	I	G	L	S	I	T	L	H	I	G	F	D	N	F	E	Q	L	L	S
Eel-1	W	V	G	D	R	Y	S	L	W	S	A	I	G	L	S	I	A	L	H	I	G	F	D	N	F	E	Q	L	L	S
Arowana-1	W	V	G	G	R	Y	S	L	W	S	A	I	G	L	S	I	A	L	H	I	G	F	E	N	F	E	Q	L	L	C
Fugu-2	W	V	G	G	R	F	S	L	W	S	A	I	G	M	A	I	A	L	H	I	G	F	D	N	F	E	K	L	L	S
Mullet-2	W	V	G	G	R	F	S	L	W	S	A	I	G	M	A	I	A	L	H	I	G	F	D	N	F	E	K	L	L	S
Smelt-2	W	V	G	G	R	F	S	L	W	S	A	I	G	M	A	I	A	L	H	I	G	F	D	N	F	E	K	L	L	S
Zebrafish-2	W	V	G	G	R	Y	S	P	W	S	A	I	G	L	S	I	A	L	H	I	G	Y	E	N	F	E	K	L	L	A
Eel-2	W	V	G	G	R	F	S	L	W	S	A	I	G	M	S	I	A	L	H	I	G	F	E	N	F	E	K	L	L	S
Arowana-2	W	V	G	G	R	Y	S	L	W	S	A	I	G	L	S	I	A	L	H	I	G	F	E	N	F	E	K	L	L	S
Sturgeon	W	V	G	G	R	Y	S	L	W	S	A	I	G	L	S	I	A	L	H	I	G	F	E	N	F	E	H	L	L	S
Gar	W	V	G	G	R	Y	S	L	W	S	A	I	G	L	S	I	A	L	H	I	G	F	E	N	F	E	R	L	L	S
Amia	W	V	G	G	R	Y	S	L	W	S	A	I	G	L	S	I	A	L	H	I	G	F	E	N	F	E	K	L	L	S
Bichir	W	V	G	G	R	Y	S	L	W	S	A	I	G	L	S	I	A	L	H	I	G	F	E	N	F	E	Q	L	L	S
Mouse	W	V	G	G	R	Y	S	L	W	S	A	I	G	L	S	I	A	L	H	V	G	F	D	H	F	E	Q	L	L	S
Rat	W	V	G	G	R	Y	S	L	W	S	A	I	G	L	S	I	A	L	H	V	G	F	D	H	F	E	Q	L	L	S
Hamster	W	V	G	G	R	Y	S	L	W	S	A	I	G	L	S	I	A	L	H	I	G	F	D	N	F	E	Q	L	L	S
Pig	W	V	G	G	R	Y	S	L	W	S	A	I	G	L	S	I	A	L	H	V	G	F	D	N	F	E	Q	L	L	S
Rabbit	W	V	G	G	R	Y	S	L	W	S	A	I	G	L	S	I	A	L	H	V	G	F	D	N	F	E	Q	L	L	S
Human	W	V	G	G	R	Y	S	L	W	S	A	I	G	L	S	I	A	L	H	V	G	F	D	N	F	E	Q	L	L	S
Snake	W	V	G	G	R	Y	S	L	W	S	A	I	G	L	S	I	A	L	H	I	G	F	D	N	F	E	K	L	L	A
Chicken	W	V	G	G	R	Y	S	L	W	S	A	I	G	L	S	I	A	L	H	I	G	F	D	N	F	E	S	L	L	A
Toad	W	V	G	G	R	Y	S	L	W	S	A	I	G	L	S	I	A	L	H	V	G	F	D	N	F	E	K	L	L	A
Hagfish	W	V	G	G	R	F	S	L	W	S	A	I	G	L	S	I	A	L	H	I	G	F	E	N	F	E	H	L	L	Q

Supplementary Fig. S2 (continued).

Position	298	299	300	301	302	303	304	305	306	307	308	309	310	311	312	313	314	315	316	317	318	319	320	321	322	323	324	325	326	327
Fugu-1	G	A	H	W	M	D	K	H	F	R	S	A	P	L	E	S	N	V	P	V	L	L	A	L	L	G	V	W	Y	I
Mullet-1	G	A	H	W	M	D	N	H	F	R	S	A	P	L	D	Q	N	V	P	V	L	L	A	V	L	G	V	W	Y	V
Smelt-1	G	A	H	W	M	D	N	H	F	H	S	A	P	L	E	K	N	V	P	V	L	L	A	M	L	G	V	W	Y	V
Zebrafish-1	G	A	H	W	M	D	N	H	F	R	S	A	P	L	G	Q	N	A	P	V	I	L	A	L	L	G	V	W	Y	V
Eel-1	G	A	H	W	M	D	N	H	F	R	R	T	P	L	E	A	N	V	P	V	L	L	A	I	L	G	V	W	Y	I
Arowana-1	G	A	H	W	M	D	N	H	F	R	S	A	P	L	E	S	N	A	P	V	L	L	A	L	L	G	I	W	Y	I
Fugu-2	G	A	H	W	M	D	N	H	F	R	T	A	P	L	D	K	N	A	P	V	L	L	A	L	L	G	I	W	Y	I
Mullet-2	G	A	H	W	M	D	N	H	F	R	S	A	P	L	D	K	N	A	P	V	L	L	A	L	L	G	I	W	Y	I
Smelt-2	G	A	H	W	M	D	N	H	F	R	T	A	P	L	D	K	N	A	P	V	L	L	A	L	L	G	I	W	Y	I
Zebrafish-2	G	A	H	W	M	D	T	H	F	R	T	A	P	V	D	Q	N	A	P	M	L	L	A	L	L	G	I	W	Y	I
Eel-2	G	A	H	W	M	D	N	H	F	R	T	A	P	L	E	K	N	A	P	V	L	L	A	M	L	G	I	W	Y	I
Arowana-2	G	A	H	W	M	D	N	H	F	R	T	A	P	L	E	S	N	A	P	M	I	L	A	L	L	G	I	W	Y	I
Sturgeon	G	A	H	W	M	D	N	H	F	R	N	T	P	L	D	K	N	V	P	V	L	L	A	L	L	G	V	W	Y	G
Gar	G	A	H	W	M	D	N	H	F	R	T	A	P	L	E	K	N	V	P	V	L	L	A	L	L	G	V	W	Y	I
Amia	G	A	H	W	M	D	N	H	F	R	T	A	P	L	E	Q	N	V	P	I	L	L	A	L	L	G	V	W	Y	I
Bichir	G	A	H	W	M	D	N	H	F	H	N	T	P	L	D	K	N	V	P	V	L	L	A	L	L	G	V	W	Y	I
Mouse	G	A	H	W	M	D	Q	H	F	L	K	T	P	L	E	K	N	A	P	V	L	L	A	L	L	G	I	W	Y	I
Rat	G	A	H	W	M	D	Q	H	F	M	K	T	P	L	D	K	N	A	P	V	L	L	A	L	L	G	I	W	Y	I
Hamster	G	A	H	W	M	D	Q	H	F	R	K	T	P	L	E	K	N	A	P	V	L	L	A	L	L	G	I	W	Y	I
Pig	G	A	H	W	M	D	Q	H	F	R	T	T	P	L	E	K	N	A	P	V	L	L	A	L	L	G	I	W	Y	I
Rabbit	G	A	H	W	M	D	Q	H	F	R	T	T	P	L	E	K	N	A	P	V	L	L	A	M	L	G	I	W	Y	I
Human	G	A	H	W	M	D	Q	H	F	R	T	T	P	L	E	K	N	A	P	V	L	L	A	L	L	G	I	W	Y	I
Snake	G	A	H	W	M	D	N	H	F	R	S	T	P	L	E	K	N	V	P	V	L	L	A	M	L	G	V	W	Y	I
Chicken	G	G	H	W	M	D	K	H	F	H	T	A	P	L	E	K	N	V	P	V	L	L	A	M	L	G	V	W	Y	I
Toad	G	A	H	W	M	D	N	H	F	C	N	T	P	L	E	N	N	I	P	V	I	L	A	M	L	G	V	W	Y	I
Hagfish	G	A	H	W	M	D	N	H	F	C	S	A	P	L	D	Q	N	V	P	V	I	M	A	L	L	R	I	W	Y	G

Position	328	329	330	331	332	333	334	335	336	337	338	339	340	341	342	343	344	345	346	347	348	349	350	351	352	353	354	355	356	357
Fugu-1	N	F	F	Q	A	E	T	Q	A	L	L	P	Y	D	Q	Y	M	H	R	F	A	A	Y	F	Q	Q	G	D	M	E
Mullet-1	N	F	F	Q	A	E	T	H	V	L	L	P	Y	D	Q	Y	L	H	R	F	A	A	Y	F	Q	Q	G	D	M	E
Smelt-1	N	F	F	Q	A	E	T	H	A	M	L	P	Y	D	Q	Y	M	H	R	F	A	A	Y	F	Q	Q	G	D	M	E
Zebrafish-1	N	F	F	Q	A	E	T	H	A	L	L	P	Y	D	Q	Y	M	H	R	F	A	A	Y	F	Q	Q	G	D	M	E
Eel-1	N	F	F	Q	A	E	T	H	A	M	L	P	Y	D	Q	Y	M	H	R	F	A	A	Y	F	Q	Q	G	D	M	E
Arowana-1	N	F	F	Q	A	E	T	H	A	L	L	P	Y	D	Q	Y	M	H	R	F	A	A	Y	F	Q	Q	G	D	M	E
Fugu-2	N	F	F	H	A	E	N	H	A	L	L	P	Y	D	Q	Y	M	H	R	F	A	A	Y	F	Q	Q	G	D	M	E
Mullet-2	N	F	F	H	A	E	T	H	A	M	L	P	Y	D	Q	Y	M	H	R	F	T	A	Y	F	Q	Q	G	D	M	E
Smelt-2	N	F	F	H	A	E	T	H	A	M	L	P	Y	D	Q	Y	M	H	R	F	T	A	Y	F	Q	Q	G	D	M	E
Zebrafish-2	N	F	F	Q	A	E	T	H	C	L	L	P	Y	D	Q	Y	M	H	R	F	A	A	Y	F	Q	Q	G	D	M	E
Eel-2	N	F	F	Q	M	E	T	H	V	M	L	P	Y	D	Q	Y	M	H	R	F	A	A	Y	F	Q	Q	G	D	M	E
Arowana-2	N	F	F	Q	A	E	T	H	A	L	L	P	Y	D	Q	Y	M	H	R	F	A	A	Y	F	Q	Q	G	D	M	E
Sturgeon	N	M	F	G	A	E	T	H	A	L	L	P	Y	D	Q	Y	M	H	R	F	A	A	Y	F	Q	Q	G	D	M	E
Gar	N	F	F	Q	A	E	T	H	A	L	L	P	Y	D	Q	Y	M	H	R	F	A	A	Y	F	Q	Q	G	D	M	E
Amia	N	F	F	Q	A	E	T	H	V	M	L	P	Y	D	Q	Y	M	H	R	F	A	A	Y	F	Q	Q	G	D	M	E
Bichir	N	L	F	G	A	E	T	H	A	L	L	P	Y	D	Q	Y	M	H	R	F	A	A	Y	F	Q	Q	G	D	M	E
Mouse	N	C	Y	G	C	E	T	H	A	L	L	P	Y	D	Q	Y	M	H	R	F	A	A	Y	F	Q	Q	G	D	M	E
Rat	N	F	Y	G	C	E	T	H	A	M	L	P	Y	D	Q	Y	M	H	R	F	A	A	Y	F	Q	Q	G	D	M	E
Hamster	N	F	Y	G	C	E	T	H	A	L	L	P	Y	D	Q	Y	M	H	R	F	A	A	Y	F	Q	Q	G	D	M	E
Pig	N	F	F	G	C	E	T	H	A	M	L	P	Y	D	Q	Y	L	H	R	F	A	A	Y	F	Q	Q	G	D	M	E
Rabbit	N	C	F	G	C	E	T	Q	A	V	L	P	Y	D	Q	Y	L	H	R	F	A	A	Y	F	Q	Q	G	D	M	E
Human	N	C	F	G	C	E	T	H	A	M	L	P	Y	D	Q	Y	L	H	R	F	A	A	Y	F	Q	Q	G	D	M	E
Snake	N	C	Y	G	T	E	T	H	A	L	L	P	Y	D	Q	Y	M	H	R	F	A	A	Y	F	Q	Q	G	D	M	E
Chicken	N	C	Y	G	C	E	T	H	A	L	L	P	Y	D	Q	Y	M	H	R	F	A	A	Y	F	Q	Q	G	D	M	E
Toad	N	F	Y	G	C	E	T	Q	A	L	L	P	Y	D	Q	Y	M	H	R	F	A	A	Y	F	Q	Q	G	D	M	E
Hagfish	N	F	Y	K	A	E	T	H	A	V	L	P	Y	D	Q	Y	L	H	R	F	T	A	Y	F	Q	Q	G	D	M	E

Supplementary Fig. S2 (continued).

Position	358	359	360	361	362	363	364	365	366	367	368	369	370	371	372	373	374	375	376	377	378	379	380	381	382	383	384	385	386	387
Fugu-1	S	N	G	K	Y	I	T	K	D	G	V	R	V	Q	Y	H	T	G	P	I	V	W	G	E	P	G	T	N	G	Q
Mullet-1	S	N	G	K	S	V	T	K	D	G	S	R	V	D	Y	N	T	G	P	I	V	W	G	E	P	G	T	N	G	Q
Smelt-1	S	N	G	K	Y	I	T	K	D	G	T	R	V	N	Y	H	T	G	P	I	V	W	G	E	P	G	T	N	G	Q
Zebrafish-1	S	N	G	K	Y	I	T	K	S	G	T	R	V	N	Y	H	T	G	P	I	V	W	G	E	P	G	T	N	G	Q
Eel-1	S	N	G	K	Y	I	T	S	Q	G	T	R	V	N	Y	H	T	G	P	I	V	W	G	E	P	G	T	N	G	Q
Arowana-1	S	N	G	K	Y	I	T	N	Q	G	T	R	V	N	H	H	T	G	P	I	V	W	G	E	P	G	T	N	G	Q
Fugu-2	S	N	G	K	Y	I	T	S	Q	G	V	R	V	N	Y	H	T	G	P	I	V	W	G	E	P	G	T	N	G	Q
Mullet-2	S	N	G	K	Y	I	T	N	H	G	A	R	V	N	Y	H	T	G	P	I	V	W	G	E	P	G	T	N	G	Q
Smelt-2	S	N	G	K	Y	I	T	K	D	G	A	R	V	N	Y	H	T	G	P	I	V	W	G	E	P	G	T	N	G	Q
Zebrafish-2	S	N	G	K	Y	I	T	T	K	G	T	R	V	N	Y	H	T	G	P	I	V	W	G	E	P	G	T	N	G	Q
Eel-2	S	N	G	K	Y	I	T	N	H	G	T	R	V	N	Y	H	T	G	P	I	V	W	G	E	P	G	T	N	G	Q
Arowana-2	S	N	G	K	Y	I	T	K	A	G	A	R	V	N	Y	H	T	G	P	I	V	W	G	E	P	G	T	N	G	Q
Sturgeon	S	N	G	K	Y	I	T	S	K	G	T	R	V	D	Y	S	T	G	P	I	V	W	G	E	P	G	T	N	G	Q
Gar	S	N	G	K	Y	I	T	S	K	G	S	R	V	N	Y	H	T	G	P	I	V	W	G	E	P	G	T	N	G	Q
Amia	S	N	G	K	Y	I	T	S	K	G	D	R	V	N	Y	H	T	G	P	I	V	W	G	E	P	G	T	N	G	Q
Bichir	S	N	G	K	Y	I	T	S	Q	G	T	R	V	N	Y	N	T	G	P	I	V	W	G	E	P	G	T	N	G	Q
Mouse	S	N	G	K	Y	I	T	K	S	G	A	R	V	D	H	Q	T	G	P	I	V	W	G	E	P	G	T	N	G	Q
Rat	S	N	G	K	Y	I	T	K	S	G	A	R	V	D	Y	Q	T	G	P	I	V	W	G	E	P	G	T	N	G	Q
Hamster	S	N	G	K	S	I	T	R	S	G	T	R	V	D	H	H	T	G	P	I	V	W	G	E	P	G	T	N	G	Q
Pig	S	N	G	K	Y	I	T	K	S	G	T	R	V	D	H	Q	T	G	P	I	V	W	G	E	P	G	T	N	G	Q
Rabbit	S	N	G	K	Y	I	T	K	S	G	A	R	V	D	H	Q	T	G	P	I	V	W	G	E	P	G	T	N	G	Q
Human	S	N	G	K	Y	I	T	K	S	G	T	R	V	D	H	Q	T	G	P	I	V	W	G	E	P	G	T	N	G	Q
Snake	S	N	G	K	Y	I	T	R	K	G	T	R	V	N	Y	N	T	G	P	I	V	W	G	E	P	G	T	N	G	Q
Chicken	S	N	G	K	Y	I	T	K	K	G	S	R	V	D	Y	N	T	G	P	I	V	W	G	E	P	G	T	N	G	Q
Toad	S	N	G	K	Y	I	T	K	T	G	A	R	V	N	Y	N	T	G	P	V	V	W	G	E	P	G	T	N	G	Q
Hagfish	S	N	G	K	Y	I	T	S	N	G	H	R	V	D	Y	S	T	G	P	I	V	W	G	E	P	G	T	N	G	Q

Position	388	389	390	391	392	393	394	395	396	397	398	399	400	401	402	403	404	405	406	407	408	409	410	411	412	413	414	415	416	417
Fugu-1	H	A	F	Y	Q	L	I	H	Q	G	T	R	M	I	P	A	D	F	L	I	P	A	Q	S	Q	H	P	I	R	N
Mullet-1	H	A	F	Y	Q	L	V	H	Q	S	T	R	L	I	P	A	D	F	L	I	P	A	Q	S	Q	H	P	I	R	N
Smelt-1	H	A	F	Y	Q	L	I	H	Q	G	T	R	M	I	P	A	D	F	L	I	P	A	Q	S	Q	H	P	I	R	D
Zebrafish-1	H	A	F	Y	Q	L	I	H	Q	G	T	R	L	I	P	A	D	F	L	I	P	A	Q	S	Q	H	P	I	R	D
Eel-1	H	A	F	Y	Q	L	I	H	Q	G	T	R	M	I	P	A	D	F	L	I	P	A	Q	T	Q	H	P	I	R	D
Arowana-1	H	A	F	Y	Q	L	I	H	Q	G	T	R	M	I	P	A	D	F	L	M	P	A	Q	T	Q	H	P	I	R	N
Fugu-2	H	A	F	Y	Q	L	I	H	Q	G	T	R	M	V	P	A	D	F	L	I	P	A	Q	S	Q	H	P	I	R	D
Mullet-2	H	A	F	Y	Q	L	I	H	Q	G	T	R	M	V	P	S	D	F	L	I	P	A	Q	T	Q	H	P	I	R	D
Smelt-2	H	A	F	Y	Q	L	I	H	Q	G	T	R	M	V	P	A	D	F	L	I	P	A	Q	S	Q	H	P	I	R	D
Zebrafish-2	H	A	F	Y	Q	L	I	H	Q	G	T	R	M	V	P	A	D	F	L	I	P	A	Q	T	Q	H	P	I	R	N
Eel-2	H	A	F	Y	Q	L	I	H	Q	G	T	R	M	V	P	A	D	F	L	I	P	V	Q	T	Q	H	P	I	R	N
Arowana-2	H	A	F	Y	Q	L	I	H	Q	G	T	R	M	V	P	A	D	F	L	I	P	A	Q	T	Q	H	P	I	R	D
Sturgeon	H	A	F	Y	Q	L	I	H	Q	G	T	R	M	I	P	A	D	F	H	I	P	V	Q	T	Q	N	P	I	R	N
Gar	H	A	F	Y	Q	L	I	H	Q	G	T	R	M	I	P	C	D	F	L	I	P	V	Q	S	Q	H	P	I	R	N
Amia	H	A	F	Y	Q	L	I	H	Q	G	T	R	M	I	P	A	D	F	L	I	P	A	Q	S	Q	N	P	I	R	S
Bichir	H	A	F	Y	Q	L	I	H	Q	G	T	R	M	V	P	A	D	F	H	I	P	V	Q	S	Q	H	P	I	R	N
Mouse	H	A	F	Y	Q	L	I	H	Q	G	T	K	M	I	P	C	D	F	L	I	P	V	Q	T	Q	H	P	I	R	K
Rat	H	A	F	Y	Q	L	I	H	Q	G	T	K	M	I	P	C	D	F	L	I	P	V	Q	T	Q	H	P	I	R	N
Hamster	H	A	F	Y	Q	L	I	H	Q	G	T	K	M	I	P	C	D	F	L	I	P	V	Q	T	Q	H	P	I	R	K
Pig	H	A	F	Y	Q	L	I	H	Q	G	T	K	M	I	P	C	D	F	L	I	P	V	Q	T	Q	H	P	I	R	K
Rabbit	H	A	F	Y	Q	L	I	H	Q	G	T	K	M	I	P	C	D	F	L	I	P	V	Q	T	Q	H	P	I	R	K
Human	H	A	F	Y	Q	L	I	H	Q	G	T	K	M	I	P	C	D	F	L	I	P	V	Q	T	Q	H	P	I	R	K
Snake	H	A	F	Y	Q	L	I	H	Q	G	T	R	M	I	P	C	D	F	L	I	P	A	Q	T	Q	H	P	I	R	N
Chicken	H	A	F	Y	Q	L	I	H	Q	G	T	R	M	I	P	C	D	F	M	I	P	V	Q	T	Q	H	P	V	R	N
Toad	H	A	F	Y	Q	L	I	H	Q	G	T	R	M	I	P	C	D	F	M	I	P	A	Q	S	Q	N	P	I	R	D
Hagfish	H	A	F	Y	Q	L	I	H	Q	G	T	R	L	V	P	A	D	F	L	I	P	V	Q	T	H	H	P	I	H	G

Supplementary Fig. S2 (continued).

Position	418	419	420	421	422	423	424	425	426	427	428	429	430	431	432	433	434	435	436	437	438	439	440	441	442	443	444	445	446	447
Fugu-1	N	L	H	H	K	I	L	V	A	N	F	L	A	Q	T	E	A	L	M	K	G	K	T	A	D	E	A	R	K	E
Mullet-1	N	L	H	H	K	I	L	V	A	N	F	L	A	Q	T	E	A	L	M	K	G	K	T	K	E	E	A	R	K	E
Smelt-1	N	L	H	H	K	I	L	M	A	N	F	L	A	Q	T	E	A	L	M	K	G	K	T	S	A	E	A	K	K	E
Zebrafish-1	N	L	H	H	K	I	L	M	A	N	F	L	A	Q	T	E	A	L	M	R	G	K	T	S	D	E	A	K	K	E
Eel-1	N	L	H	H	K	I	L	I	A	N	F	L	A	Q	T	E	A	L	M	R	G	K	T	P	E	E	A	R	K	E
Arowana-1	N	L	H	H	K	I	L	T	A	N	F	L	A	Q	T	E	A	L	M	K	G	K	K	P	E	E	A	R	K	E
Fugu-2	N	L	H	H	K	I	L	V	A	N	F	L	A	Q	T	E	A	L	M	R	G	K	T	T	E	E	A	R	K	E
Mullet-2	N	L	H	H	K	I	L	L	A	N	F	L	A	Q	T	E	A	L	M	K	G	K	T	T	E	E	A	R	K	E
Smelt-2	N	L	H	H	K	I	L	L	A	N	F	L	A	Q	T	E	A	L	M	K	G	K	T	T	E	E	A	K	K	E
Zebrafish-2	S	L	H	H	K	I	L	L	A	N	F	L	A	Q	T	E	A	L	M	K	G	K	T	T	E	E	A	K	K	E
Eel-2	N	L	H	H	K	I	L	M	A	N	F	L	A	Q	T	E	A	L	M	K	G	K	T	T	E	E	A	K	K	E
Arowana-2	N	L	H	H	K	I	L	M	A	N	F	L	A	Q	T	E	A	L	M	K	G	K	T	A	E	E	A	R	K	E
Sturgeon	N	L	H	H	K	I	L	M	A	N	F	L	A	Q	T	E	A	L	M	K	G	K	T	T	E	E	A	R	K	E
Gar	N	L	H	H	K	I	L	L	A	N	F	L	A	Q	T	E	A	L	M	K	G	K	T	T	E	E	A	R	K	E
Amia	N	L	H	H	K	I	L	L	A	N	F	L	A	Q	T	E	A	L	M	K	G	K	S	S	D	E	A	Q	K	E
Bichir	N	L	H	H	K	I	L	L	A	N	F	L	A	Q	T	E	A	L	M	K	G	K	T	S	E	E	A	R	K	E
Mouse	G	L	H	H	K	I	L	L	A	N	F	L	A	Q	T	E	A	L	M	K	G	K	L	P	E	E	A	R	K	E
Rat	G	L	H	H	K	I	L	L	A	N	F	L	A	Q	T	E	A	L	M	K	G	K	S	P	E	E	A	R	K	E
Hamster	G	L	H	H	K	I	L	L	A	N	F	L	A	Q	T	E	A	L	M	K	G	K	S	N	E	E	A	K	K	E
Pig	G	L	H	H	K	I	L	L	A	N	F	L	A	Q	T	E	A	L	M	K	G	K	S	T	E	E	A	R	K	E
Rabbit	G	L	H	H	K	I	L	L	A	N	F	L	A	Q	T	E	A	L	M	K	G	K	S	T	E	E	A	R	K	E
Human	G	L	H	H	K	I	L	L	A	N	F	L	A	Q	T	E	A	L	M	R	G	K	S	T	E	E	A	R	K	E
Snake	G	L	H	H	K	I	L	L	A	N	F	L	A	Q	T	E	A	L	M	K	G	K	T	T	E	E	A	R	Q	E
Chicken	G	L	H	H	K	I	L	L	A	N	F	L	A	Q	T	E	A	L	M	K	G	K	T	A	D	E	A	R	K	E
Toad	G	L	H	H	K	I	L	M	A	N	F	L	A	Q	T	E	A	L	M	K	G	K	S	T	E	E	A	K	A	E
Hagfish	G	M	H	H	Q	I	L	M	A	N	F	L	A	Q	T	E	A	L	M	K	G	K	T	E	A	E	A	R	K	E

Position	448	449	450	451	452	453	454	455	456	457	458	459	460	461	462	463	464	465	466	467	468	469	470	471	472	473	474	475	476	477
Fugu-1	L	D	A	A	G	L	K	A	D	A	I	E	K	L	L	P	H	K	V	F	E	G	N	K	P	S	N	S	I	V
Mullet-1	L	E	A	G	G	L	K	G	G	A	L	E	E	L	L	P	H	K	V	F	E	G	N	K	P	S	N	S	I	V
Smelt-1	L	E	A	T	G	L	A	G	P	A	L	E	N	L	L	P	H	K	V	F	Q	G	N	K	P	S	N	S	I	V
Zebrafish-1	L	Q	A	S	G	L	S	G	D	S	L	E	K	L	L	P	H	K	V	F	Q	G	N	K	P	S	N	S	I	I
Eel-1	L	E	A	S	G	M	S	G	E	P	L	E	K	L	L	P	H	K	V	F	E	G	N	K	P	T	N	S	I	V
Arowana-1	L	E	A	S	G	L	S	G	E	A	L	E	K	L	L	P	H	K	V	F	E	G	N	K	P	T	D	S	I	V
Fugu-2	L	E	S	G	G	L	N	G	E	A	L	D	K	I	L	P	H	K	V	F	Q	G	N	K	P	T	N	S	I	I
Mullet-2	L	E	A	S	G	L	S	G	E	A	L	E	K	I	L	P	H	K	V	F	Q	G	N	R	P	T	N	S	I	I
Smelt-2	L	E	A	A	G	L	T	G	E	A	L	D	K	L	L	P	H	K	V	F	Q	G	N	K	P	T	N	S	I	I
Zebrafish-2	L	E	A	G	G	L	S	G	D	N	L	E	K	I	L	P	H	K	V	F	Q	G	N	K	P	T	D	S	I	V
Eel-2	L	E	A	G	G	L	S	G	E	A	L	E	K	L	L	P	H	K	V	F	P	G	N	K	P	T	N	S	I	I
Arowana-2	L	E	A	G	G	L	S	G	E	A	L	E	K	L	L	P	H	K	V	F	E	G	N	K	P	T	N	S	V	V
Sturgeon	L	E	G	A	G	L	S	G	E	A	L	E	N	L	L	P	H	K	V	F	E	G	N	K	P	T	N	S	I	V
Gar	L	E	G	A	G	M	S	G	E	P	L	E	K	L	L	P	H	K	V	F	E	G	N	K	P	T	N	S	I	V
Amia	L	E	G	A	G	M	S	G	E	A	L	E	K	L	L	P	H	K	V	F	Q	G	N	K	P	T	N	S	M	V
Bichir	L	E	G	A	G	L	S	G	E	A	L	E	K	L	L	P	H	K	V	F	E	G	N	R	P	T	N	S	M	V
Mouse	L	Q	A	A	G	K	S	P	E	D	L	E	K	L	L	P	H	K	V	F	E	G	N	R	P	T	N	S	I	V
Rat	L	Q	A	A	G	K	S	P	E	E	L	E	K	L	L	P	H	K	V	F	E	G	N	R	P	T	N	S	I	V
Hamster	L	Q	A	A	G	K	S	P	E	D	L	E	K	L	L	P	H	K	V	F	E	G	N	R	P	T	N	S	I	V
Pig	L	Q	A	A	G	K	S	P	E	D	F	E	K	L	L	P	H	K	V	F	E	G	N	R	P	T	N	S	I	V
Rabbit	L	Q	A	A	G	K	S	P	E	D	L	M	K	L	L	P	H	K	V	F	E	G	N	R	P	T	N	S	I	V
Human	L	Q	A	A	G	K	S	P	E	D	L	E	R	L	L	P	H	K	V	F	E	G	N	R	P	T	N	S	I	V
Snake	L	Q	A	A	G	L	S	G	E	A	L	E	K	L	L	P	H	K	V	F	E	G	N	R	P	T	N	S	I	V
Chicken	L	Q	A	A	G	L	S	G	D	A	L	E	K	L	L	P	H	K	V	F	E	G	N	R	P	T	N	S	I	M
Toad	L	Q	A	S	G	L	S	G	E	A	L	E	K	L	L	P	H	K	V	F	E	G	N	R	P	T	N	S	I	V
Hagfish	L	Q	A	A	G	K	S	G	D	E	L	E	K	L	L	P	H	K	V	F	E	G	N	R	P	T	N	S	L	I

Supplementary Fig. S2 (continued).

Position	478	479	480	481	482	483	484	485	486	487	488	489	490	491	492	493	494	495	496	497	498	499	500	501	502	503	504	505	506	507
Fugu-1	F	K	K	L	S	P	F	T	L	G	A	L	I	A	M	Y	E	H	K	I	F	V	Q	G	V	I	W	D	I	N
Mullet-1	F	K	K	L	T	P	F	I	L	G	S	L	V	A	M	Y	E	H	K	I	F	V	Q	G	V	M	W	N	I	N
Smelt-1	F	K	K	L	S	P	F	M	L	G	A	L	V	A	M	Y	E	H	K	I	F	V	Q	G	V	V	W	D	I	N
Zebrafish-1	F	K	K	L	T	P	F	M	L	G	A	L	V	A	M	Y	E	H	K	I	F	V	Q	G	V	M	W	N	I	N
Eel-1	F	K	K	L	T	P	F	I	L	G	A	L	V	A	M	Y	E	H	K	I	F	V	Q	G	V	M	W	E	I	N
Arowana-1	F	K	K	L	T	P	F	V	L	G	S	L	V	A	M	Y	E	H	K	I	F	V	Q	G	V	I	W	E	I	N
Fugu-2	F	R	K	L	S	P	Y	T	L	G	A	L	T	A	M	Y	E	H	K	T	F	I	Q	G	L	L	W	E	I	N
Mullet-2	F	K	K	L	T	P	F	T	L	G	A	L	I	A	M	Y	E	H	K	I	F	V	Q	G	V	M	W	E	I	N
Smelt-2	F	K	K	L	N	P	Y	T	L	G	A	L	I	A	M	Y	E	H	K	I	F	V	Q	G	T	M	W	E	I	N
Zebrafish-2	F	K	K	L	S	P	F	T	L	G	V	L	I	A	M	Y	E	H	K	I	F	I	Q	G	V	M	W	E	I	N
Eel-2	F	K	K	L	T	P	F	I	L	G	V	L	I	A	M	Y	E	H	K	I	F	L	Q	G	T	I	W	E	I	N
Arowana-2	F	K	K	L	T	P	Y	I	L	G	V	L	I	A	M	Y	E	H	K	I	F	V	Q	G	L	M	W	E	I	N
Sturgeon	F	K	K	L	T	P	F	I	L	G	A	L	I	A	M	Y	E	H	K	I	F	V	Q	G	V	I	W	D	I	N
Gar	F	K	K	L	T	P	F	M	L	G	A	L	I	A	M	Y	E	H	K	I	F	M	Q	G	V	I	W	E	I	N
Amia	F	T	K	L	N	P	F	M	L	G	A	L	I	A	M	Y	E	H	K	I	F	V	Q	G	V	I	W	D	I	N
Bichir	F	K	K	L	T	P	F	I	L	G	A	L	I	A	M	Y	E	H	K	I	F	V	Q	G	V	I	W	D	I	N
Mouse	F	T	K	L	T	P	F	I	L	G	A	L	I	A	M	Y	E	H	K	I	F	V	Q	G	I	M	W	D	I	N
Rat	F	T	K	L	T	P	F	I	L	G	A	L	I	A	M	Y	E	H	K	I	F	V	Q	G	I	I	W	D	I	N
Hamster	F	T	K	L	T	P	F	I	L	G	A	L	I	A	L	Y	E	H	K	I	F	V	Q	G	V	I	W	D	I	N
Pig	F	T	K	L	T	P	F	I	L	G	A	L	I	A	M	Y	E	H	K	I	F	V	Q	G	V	I	W	D	I	N
Rabbit	F	T	K	L	T	P	F	I	L	G	A	L	I	A	M	Y	E	H	K	I	F	V	Q	G	V	V	W	D	I	N
Human	F	T	K	L	T	P	F	M	L	G	A	L	V	A	M	Y	E	H	K	I	F	V	Q	G	I	I	W	D	I	N
Snake	F	T	K	L	T	P	F	I	L	G	A	L	I	A	M	Y	E	R	K	I	F	V	Q	G	I	V	W	D	I	N
Chicken	F	T	K	L	N	P	F	T	L	G	A	I	I	A	M	Y	E	H	K	I	F	V	Q	G	V	V	W	D	I	N
Toad	F	G	K	L	S	P	F	I	L	G	A	L	I	A	M	Y	E	H	K	I	F	V	Q	G	V	V	W	D	I	N
Hagfish	F	K	K	L	T	P	F	T	L	G	V	L	I	A	L	Y	E	H	K	I	F	V	Q	G	V	I	W	D	I	N

Position	508	509	510	511	512	513	514	515	516	517	518	519	520	521	522	523	524	525	526	527	528	529	530	531	532	533	534	535	536	537
Fugu-1	S	Y	D	Q	W	G	V	E	L	G	K	Q	L	A	K	K	I	E	P	E	L	Q	D	D	S	A	V	S	S	H
Mullet-1	S	Y	D	Q	W	G	V	E	L	G	K	Q	L	A	K	K	I	E	P	E	L	Q	D	D	S	E	V	T	S	H
Smelt-1	S	Y	D	Q	W	G	V	E	L	G	K	Q	L	A	K	K	I	E	P	E	L	Q	D	S	S	E	V	H	S	H
Zebrafish-1	S	Y	D	Q	W	G	V	E	L	G	K	Q	L	A	K	K	I	E	P	E	L	Q	D	D	A	E	V	H	S	H
Eel-1	S	Y	D	Q	W	G	V	E	L	G	K	Q	L	A	K	K	I	E	P	E	L	Q	D	D	C	E	V	Q	T	H
Arowana-1	S	Y	D	Q	W	G	V	E	L	G	K	Q	L	A	K	R	I	E	P	E	L	Q	D	A	A	E	V	H	S	H
Fugu-2	S	F	D	Q	W	G	V	E	L	G	K	Q	L	A	K	K	I	E	P	E	L	K	D	A	T	E	V	H	S	H
Mullet-2	S	F	D	Q	W	G	V	E	L	G	K	Q	L	A	K	K	I	E	P	E	L	K	D	A	A	E	V	S	S	H
Smelt-2	S	F	D	Q	W	G	V	E	L	G	K	Q	L	A	K	Q	I	E	P	Q	L	Q	D	K	T	E	V	K	S	H
Zebrafish-2	S	F	D	Q	W	G	V	E	L	G	K	Q	L	A	K	K	I	E	P	E	L	Q	D	S	A	Q	V	S	S	H
Eel-2	S	F	D	Q	W	G	V	E	L	G	K	Q	L	A	K	K	I	E	P	E	L	Q	D	T	T	E	V	H	S	H
Arowana-2	S	F	D	Q	W	G	V	E	L	G	K	Q	L	A	K	K	I	E	A	E	L	K	D	A	A	E	V	T	S	H
Sturgeon	S	Y	D	Q	W	G	V	E	L	G	K	Q	L	A	K	Q	I	E	P	E	L	Q	D	N	S	E	V	T	S	H
Gar	S	Y	D	Q	W	G	V	E	L	G	K	Q	L	A	K	K	I	E	P	E	L	Q	D	A	G	E	V	T	S	H
Amia	S	Y	D	Q	W	G	V	E	L	G	K	Q	L	A	K	K	I	E	P	E	L	Q	D	A	S	E	V	T	S	H
Bichir	S	Y	D	Q	W	G	V	E	L	G	K	Q	L	A	K	K	I	E	P	E	L	V	D	A	S	E	V	T	S	H
Mouse	S	F	D	Q	W	G	V	E	L	G	K	Q	L	A	K	K	I	E	P	E	L	E	G	S	S	A	V	T	S	H
Rat	S	F	D	Q	W	G	V	E	L	G	K	Q	L	A	K	K	I	E	P	E	L	D	G	S	S	A	V	T	S	H
Hamster	S	F	D	Q	W	G	V	E	L	G	K	Q	L	A	K	N	I	E	P	E	L	D	G	S	A	P	V	T	S	H
Pig	S	F	D	Q	W	G	V	E	L	G	K	Q	L	A	K	K	I	E	P	E	L	D	G	S	S	P	V	T	S	H
Rabbit	S	F	D	Q	W	G	V	E	L	G	K	Q	L	A	K	K	I	E	P	E	L	D	G	S	S	P	V	T	S	H
Human	S	F	D	Q	W	G	V	E	L	G	K	Q	L	A	K	K	I	E	P	E	L	D	G	S	A	Q	V	T	S	H
Snake	S	Y	D	Q	W	G	V	E	L	G	K	Q	L	A	K	K	I	E	P	E	L	E	T	D	A	A	V	T	S	H
Chicken	S	Y	D	K	C	R	V	E	L	G	K	Q	L	A	K	K	I	E	P	E	L	E	S	D	A	P	V	T	S	H
Toad	S	Y	D	Q	W	G	V	E	L	G	K	Q	L	A	K	K	I	E	P	E	L	E	S	N	A	P	V	T	S	H
Hagfish	S	Y	D	Q	W	G	V	E	L	G	K	Q	L	A	K	A	I	E	P	E	L	A	T	P	A	T	V	S	S	H

Supplementary Fig. S2 (continued).

Position	538	539	540	541	542	543	544	545	546	547	548	549	550	551	552	553	554	555	556	557	558
Fugu-1	D	S	S	T	N	G	L	I	N	F	L	K	K	N	F	-	-	-	-	-	-
Mullet-1	D	S	S	T	N	G	L	I	N	F	L	K	K	N	F	A	*	-	-	-	-
Smelt-1	D	S	S	T	N	G	L	I	N	F	L	K	K	N	S	A	*	-	-	-	-
Zebrafish-1	D	S	S	T	N	G	L	I	G	F	F	K	K	N	R	F	*	-	-	-	-
Eel-1	D	S	S	T	N	G	L	I	G	F	I	K	K	N	Y	A	*	-	-	-	-
Arowana-1	D	S	S	T	N	G	L	I	N	F	F	K	K	N	Y	A	*	-	-	-	-
Fugu-2	D	S	S	T	N	G	L	I	N	F	L	K	K	N	F	A	*	-	-	-	-
Mullet-2	D	S	S	T	N	G	L	I	N	F	L	K	K	N	F	A	*	-	-	-	-
Smelt-2	D	S	S	T	N	G	L	I	N	F	L	K	K	N	F	-	*	-	-	-	-
Zebrafish-2	D	S	S	T	N	G	L	I	N	F	L	K	N	N	F	A	*	-	-	-	-
Eel-2	D	S	S	T	N	G	L	I	N	F	I	K	K	N	V	A	*	-	-	-	-
Arowana-2	D	S	S	T	N	G	L	I	N	F	I	K	K	N	V	A	*	-	-	-	-
Sturgeon	D	C	S	T	N	G	L	I	N	F	I	K	K	N	Q	A	*	-	-	-	-
Gar	D	G	S	T	N	G	L	I	S	F	I	K	K	N	S	A	*	-	-	-	-
Amia	D	S	S	T	N	G	L	I	G	F	I	K	K	N	S	A	*	-	-	-	-
Bichir	D	C	S	T	N	G	L	I	S	F	I	K	K	N	S	A	*	-	-	-	-
Mouse	D	S	S	T	N	G	L	I	S	F	I	K	Q	Q	R	D	T	K	L	E	*
Rat	D	S	S	T	N	G	L	I	G	F	I	K	L	Q	R	D	T	K	I	D	*
Hamster	D	S	S	T	N	G	L	I	K	F	I	K	Q	Q	R	D	I	R	I	E	*
Pig	D	S	S	T	N	G	L	I	N	F	I	K	Q	E	R	E	A	R	S	Q	*
Rabbit	D	S	S	T	N	G	L	I	N	F	I	K	Q	Q	R	E	A	K	I	Q	*
Human	D	A	S	T	N	G	L	I	N	F	I	K	Q	Q	R	E	A	R	V	Q	*
Snake	D	A	S	T	N	G	L	I	A	F	I	K	K	N	R	A	*	-	-	-	-
Chicken	D	S	S	T	N	G	L	I	S	F	I	K	K	H	R	A	*	-	-	-	-
Toad	D	S	S	T	N	G	L	I	N	F	Y	K	A	H	R	L	*	-	-	-	-
Hagfish	D	S	S	T	N	G	L	I	N	F	I	K	K	H	R	A	*	-	-	-	-

Supplementary Fig. S2 (continued)

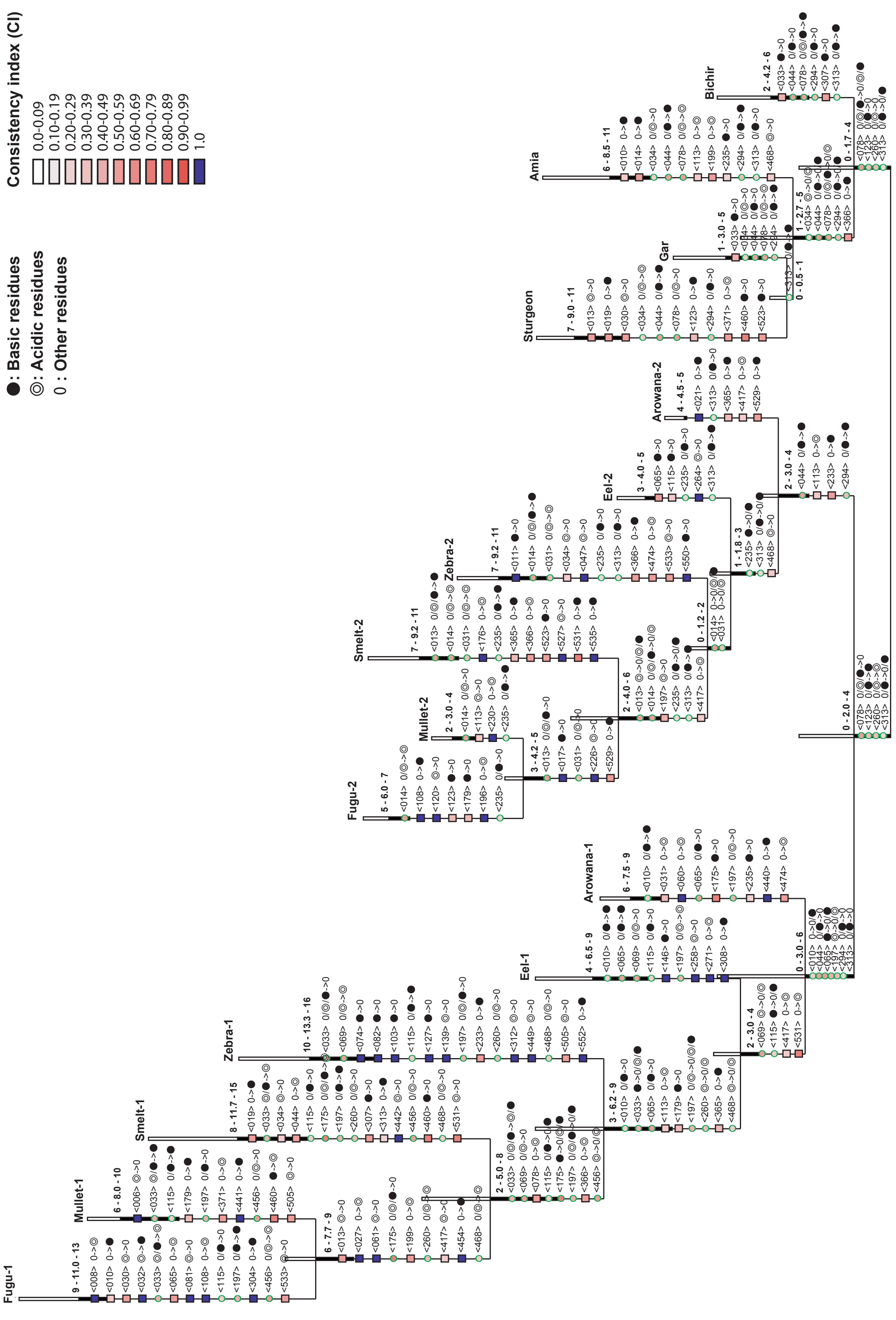

Sato and Nishida, Supplementary Fig. S3.

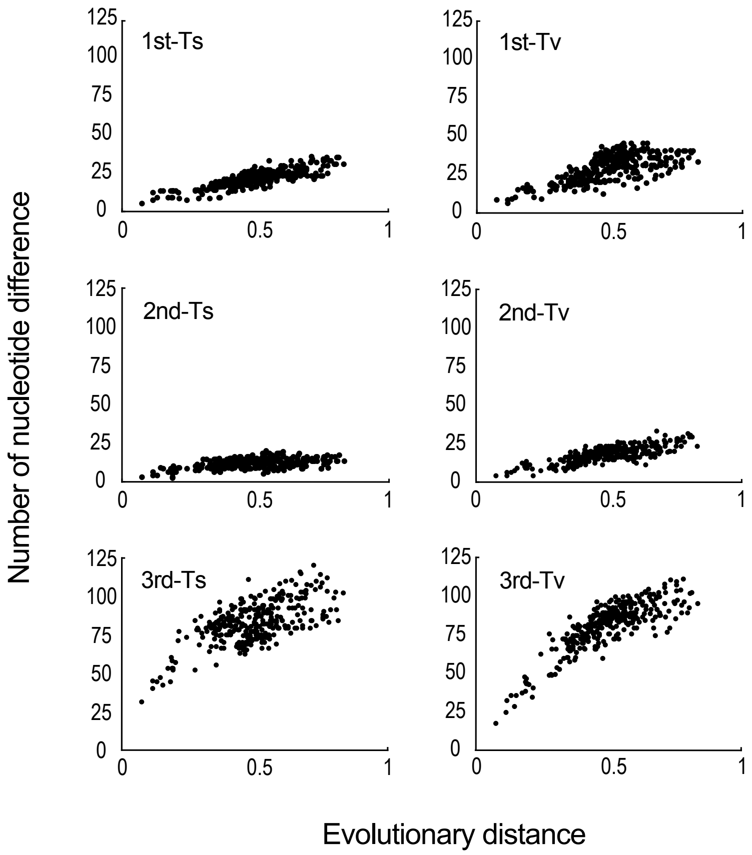

Sato and Nishida, Supplementary Fig. S4.

Non-teleost fishes

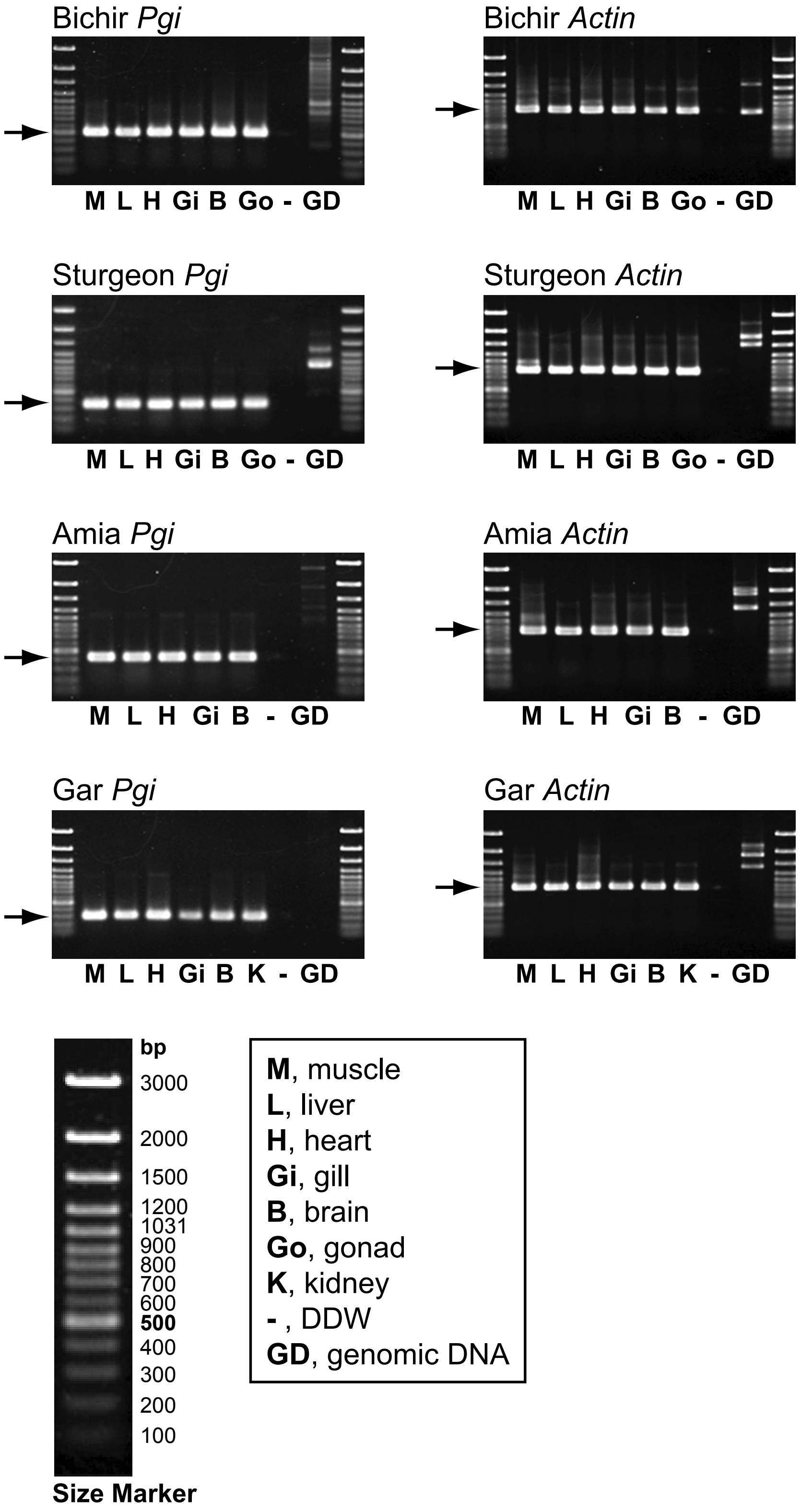

Sato and Nishida, Supplementary Fig. S5 (to be continued).

Teleost fishes

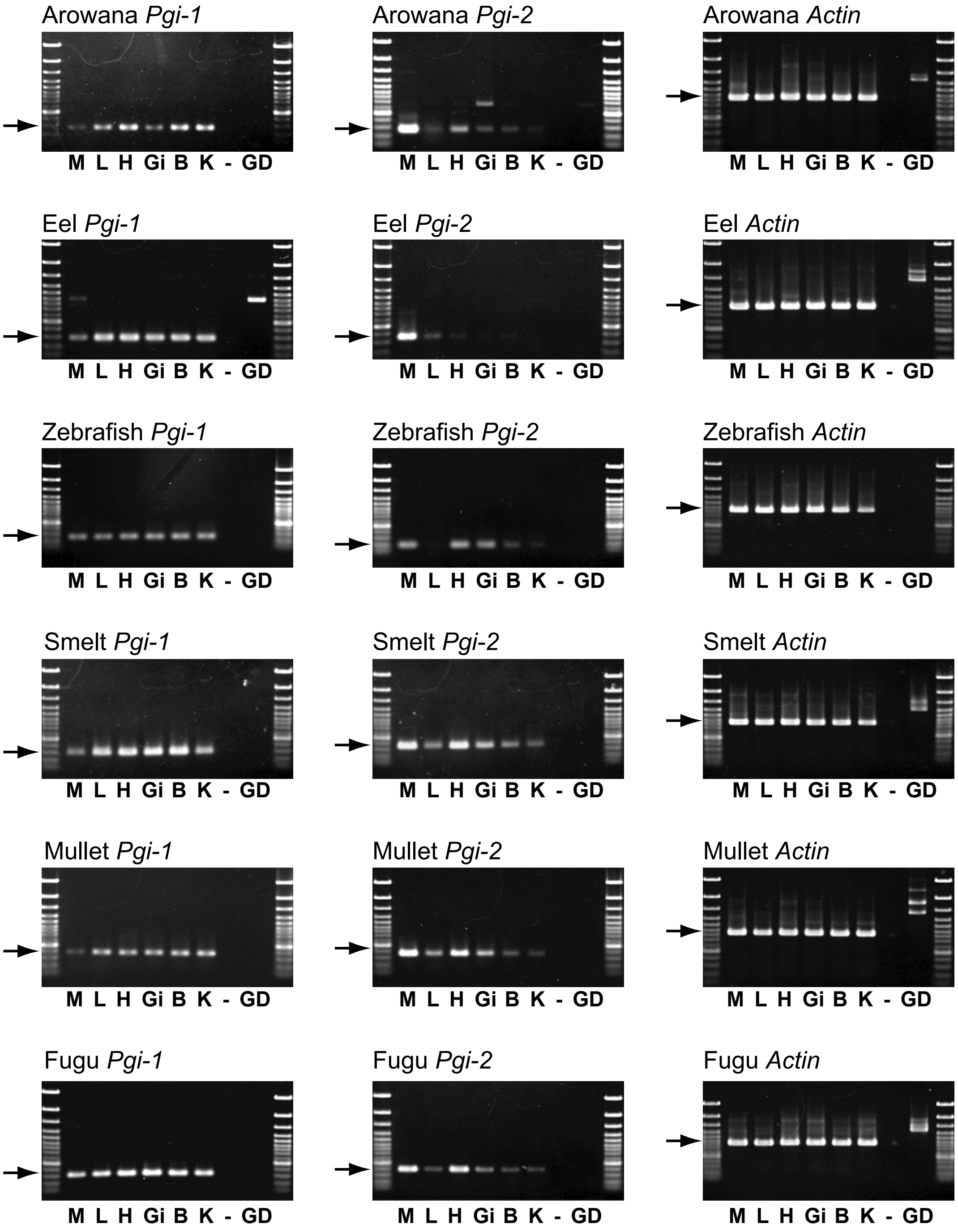

Sato and Nishida, Supplementary Fig. S5 (continued).

Supplementary References

1. Lee JH, Jeffery CJ: **The crystal structure of rabbit phosphoglucose isomerase complexed with D-sorbitol-6-phosphate, an analog of the open chain form of D-glucose-6-phosphate.** *Protein Sci* 2005, 14: 727-734

2. Inoue JG, Miya M, Tsukamoto K, Nishida M: **Basal actinopterygian relationships: a mitogenomic perspective on the phylogeny of the ''ancient fish''.** *Mol Phylogenet Evol* 2003, 26: 110-120

3. Maddison WP, Maddison DR: *MacClade, ver. 4.06. OSX.* Sinauer, Sunderland, Massachusetts 2003

4. Swofford DL: *PAUP*. Phylogenetic analysis using parsimony (*and other methods), version 4.0b10 (Alvitec).* Sinauer, Sunderland, Massachusetts 2002

5. Tamura K, Nei M: **Estimation of the number of nucleotide substitutions in the control region of mitochondrial DNA in humans and chimpanzees.** *Mol Biol Evol* 1993, 10: 512-526

6. Posada D, Crandall KA: **MODEL TEST: testing the model of DNA substitution.** *Bioinformatics Appl Note* 1998, 14: 817-818

Appendix

The ancestral nucleotide sequences (node#1—#15 of Figure 2C in the main manuscript, shown in *fasta* format) inferred by the maximum likelihood method.

```
>node#1
CTCACCAGCGACCCAAACTTCCAGAAGCTGGAGCAATGGTACAAAGCCAACGGCGGCAGCCTCA
ACATGAGGCAGATGTTTGAGGCCGACAAGGACAGGTTCAGCAAGTTCAGCACAACCCTGAAAAC
CGATGATGGAGACATCCTGATCGATTATTCAAAGAACCTCATCACTGAGGAAGTCATGAAGATGC
TGTTTGATCTGGCAAAGTCGAGGGGAGTGGAGGAAGCCAGGGAGAGGATGTTCACTGGAGAGA
AGATCAACTTCACTGAGAACCGAGCTGTGCTCCACATTGCCCTGAGGAACCGCTCCAACACTCCC
ATCCACGTGGACGGCAAGGACGTCATGCCTGAGGTCAACAATGTCCTGGAGAAGATGAAGGGCT
TCTGTCAGAAAGTGCGCAGTGGAGAGTGGAAGGGCTACACTGGAAAAGCCATCACTGATGTTGT
CAATATTGGCATTGGTGGATCTGACCTGGGCCCCTTGATGGTGACTGAAGCTCTGAAGCCCTACT
CCAAGGGAGGACCCCGGGTCTGGTTTGTCTCCAACATCGATGGCACTCACATGGCCAAGACCCT
GGCTGAGCTGAACCCTGAGACCACCCTGTTCATCATTGCATCCAAGACCTTCACCACCCAGGAG
ACCATCACCAATGCGGAGTCCGCCAAGGAGTGGTTCTTGCAGACAGCTGGAGATAAATCAGCTG
TGGCGAAGCACTTTGTGGCTCTCTCCACCAATGCTCCCAAAGTGAAGGATTTTGGGATTGACACG
AACAACATGTTTGAGTTCTGGGATTGGGTTGGTGGCCGTTATTCCCTGTGGTCAGCTATTGGCTT
GTCCATTGCTCTGCACATCGGCTTTGAGAACTTTGAGCAGCTGCTGAGTGGAGCTCACTGGATG
GACAATCACTTCCGCACCGCCCCCCTGGAGAAGAATGTCCCCGTGCTGCTGGCCCTGCTTGGGG
TCTGGTATATCAACTTCTTCCAGGCGGAGACCCACGCCCTGCTACCCTACGACCAGTACATGCAC
CGCTTTGCTGCCTACTTCCAGCAGGGTGACATGGAGTCCAATGGGAAGTACATCACCAGTAAAG
GCACCCGTGTGAACTACAACACTGGGCCCATCGTGTGGGGAGAGCCTGGGACCAATGGGCAGC
ATGCCTTCTACCAGCTCATCCATCAAGGAACACGCATGGTTCCTGCTGACTTCCTCATCCCTGTC
CAGACTCAGCATCCCATCAGGAACAACCTGCACCACAAGATCCTGCTGGCTAACTTCCTGGCCCA
GACTGAGGCCCTGATGAAGGGGAAGACCACAGAGGAGGCCCGGAAGGAGCTTGAGGGGGCAG
GCCTGAGTGGAGAGGCCCTGGAGAAACTGCTGCCTCACAAGGTCTTCGAAGGAAACCGGCCAA
CAAACTCCATTGTCTTTAAGAAGCTGACTCCCTTCATTCTTGGAGCACTGATTGCCATGTATGAAC
ACAAGATCTTCGTGCAGGGTGTCATCTGGGACATCAACAGCTATGACCAGTGGGGAGTGGAACT
GGGGAAACAGCTGGCCAAGAAAATTGAGCCCGAGCTGGAGGATGCTAGCGAGGTGACCTCACA
CGACTGCTCAACCAATGGGCTGATCAGCTTCATCAAGAAGAACAGT

>node#2
CTCACCAGCGACCCCAACTTCCAGAAGCTGGAGCAATGGTACAAAGCCAACGCCGGCAGCCTCA
ACATGAGGCAGATGTTTGAGGCCGATAAGGAGAGGTTCAGCAAGTTCAGTACAACCCTGAAAAC
CGATGATGGAGACATCCTGATCGATTATTCAAAGAACCTCATCACTGAGGAAGTCATGAAGATGC
TGTTCGATCTGGCAAAGTCGAGGGGAGTGGAGGAAGCCAGGGAGAGGATGTTCACTGGAGAGA
AGATCAACTTCACTGAGGGCCGAGCTGTGCTCCACATTGCCCTGAGGAACCGCTCCAACACTCC
CATCCACGTGGACGGCAAGGACGTCATGCCTGAGGTCAACAACGTCCTGGAGAAGATGAAGGG
CTTCTGTCAGAAAGTGCGCAGTGGAGAGTGGAAGGGCTACACTGGAAAAGCCATCACTGATGTT
GTCAACATTGGCATTGGTGGATCTGACCTGGGCCCCTTGATGGTGACTGAAGCTCTGAAGCCCT
ACTCCAAGGGAGGACCCCGGGTCTGGTTTGTCTCCAACATCGATGGCACTCACATGGCCAAGAC
CCTGGCTGAGCTGAACCCTGAGACCACCCTCTTCATCATTGCATCCAAGACCTTCACCACCCAGG
AGACCATCACCAATGCAGAGTCCGCCAAGGAGTGGTTCCTGCAGAAAGCTGGAGATAAATCTGC
TGTGGCCAAGCACTTTGTGGCTCTCTCCACCAATGCTCCCAAAGTGAAGGATTTCGGGATTGACA
CGAACAACATGTTTGAGTTCTGGGATTGGGTTGGTGGCCGTTATTCCCTGTGGTCAGCTATTGGC
TTGTCCATTGCTCTGCACATCGGCTTTGAGAACTTTGAGCAGCTGCTGAGTGGAGCTCACTGGAT
GGACAATCACTTCCGCACCGCCCCCCTGGAGAAGAACGTCCCCGTCCTGCTGGCCCTGCTGGG
GGTCTGGTACATCAACTTCTTCCAGGCGGAGACCCACGCCCTGCTGCCCTACGACCAGTACATG
CACCGCTTTGCTGCCTACTTCCAGCAGGGGGACATGGAGTCCAATGGGAAGTACATCACCAGTA
AAGGCACCCGTGTGAACTACCACACTGGGCCCATCGTGTGGGGAGAGCCTGGGACCAATGGGC
AGCATGCCTTCTACCAGCTCATCCATCAAGGAACACGCATGATTCCTGCTGACTTCCTCATCCCT
GTCCAGACTCAGCACCCCATCAGGAACAACCTGCACCACAAGATCCTGCTGGCTAACTTCCTGG
```

CCCAGACTGAGGCCCTGATGAAGGGGAAGACCACAGAGGAGGCCCGGAAGGAGCTGGAGGGG
GCAGGCCTGAGTGGAGAGGCCCTGGAGAAACTGCTGCCTCACAAGGTCTTCGAAGGAAACAAG
CCAACCAACTCCATTGTCTTTAAGAAGCTGACTCCCTTCATTCTTGGAGCACTGATTGCCATGTAT
GAACACAAGATCTTCGTGCAGGGTGTCATCTGGGACATCAACAGCTATGACCAGTGGGGAGTGG
AACTGGGGAAACAGCTGGCCAAGAAAATCGAGCCCGAGCTGCAGGATGCTAGCGAGGTGACCT
CACACGACTGCTCGACCAATGGGCTGATCAGCTTCATCAAGAAGAACAGT

>node#3
CTCACCAGCGACCCCAACTTCCAGAAGCTGGAGCAATGGTACAAAGCCAACGCCGGCAGCCTCA
ACATGAGGCAGATGTTTGAGGCCGATAAGGAGAGGTTCAGCAAGTTCAGTACAACCCTTAAAACC
GATGATGGAGACATCCTGATCGATTATTCAAAGAACCTCATCACTGAGGAAGTCATGAAGATGCT
GTTCGATCTGGCAAAGTCGAGGGGAGTGGAGGAAGCCAGGGAGAGGATGTTCACTGGAGAGAA
GATCAACTTCACTGAGGGCCGAGCTGTGCTCCACATTGCCCTGAGGAACCGCTCCAACACTCCC
ATCCACGTGGACGGCAAGGACGTCATGCCTGAGGTCAACAACGTCCTGGAGAAGATGAAGGGCT
TCTGTCAGAAAGTGCGCAGTGGAGAGTGGAAGGGCTACACTGGAAAAGCCATCACTGATGTTGT
CAACATTGGCATTGGTGGATCTGACCTGGGCCCCTTGATGGTGACTGAAGCTCTGAAGCCCTACT
CCAAGGGAGGACCCCGGGTCTGGTTTGTCTCCAACATCGATGGCACTCACATGGCCAAGACCCT
GGCTGAGCTGAACCCTGAGACCACCCTCTTCATCATTGCATCCAAGACCTTCACCACCCAGGAGA
CCATCACCAATGCAGAGTCCGCCAAGGAGTGGTTCCTGCAGAAAGCTGGAGATAAATCTGCTGT
GGCCAAGCACTTTGTGGCTCTCTCCACCAATGCTCCCAAAGTGAAGGATTTCGGGATTGACACGA
ACAACATGTTTGAGTTCTGGGATTGGGTTGGTGGCCGTTATTCCCTGTGGTCAGCTATTGGCTTG
TCCATTGCTCTGCACATCGGCTTTGAGAACTTTGAGCAGCTGCTGAGTGGAGCTCACTGGATGGA
CAATCACTTCCGCACCGCCCCCCTGGAGAAGAACGTCCCCGTCCTGCTGGCCCTGCTGGGGGT
CTGGTACATCAACTTCTTCCAGGCGGAGACCCACGCCCTGCTGCCCTACGACCAGTACATGCAC
CGCTTTGCTGCCTACTTCCAGCAGGGGGACATGGAGTCCAATGGGAAGTACATCACCAGTAAAG
GCACCCGTGTGAACTACCACACTGGGCCCATCGTGTGGGGAGAGCCTGGGACCAATGGGCAGC
ATGCCTTCTACCAGCTCATCCATCAAGGAACACGCATGATTCCTGCTGACTTCCTCATCCCTGTC
CAGACTCAGCACCCCATCAGGAACAACCTGCACCACAAGATCCTGCTGGCTAACTTCCTGGCCC
AGACTGAGGCCCTGATGAAGGGGAAGACCACAGAGGAGGCCCGGAAGGAGCTGGAGGGGGCA
GGCCTGAGTGGAGAGGCCCTGGAGAAACTGCTGCCTCACAAGGTCTTCGAAGGAAACAAGCCAA
CCAACTCCATTGTCTTTAAGAAGCTGACTCCCTTCATGCTTGGAGCACTGATTGCCATGTATGAAC
ACAAGATCTTCGTGCAGGGTGTCATCTGGGACATCAACAGCTATGACCAGTGGGGAGTGGAACT
GGGGAAACAGCTGGCCAAGAAAATCGAGCCCGAGCTGCAGGATGCTAGCGAGGTGACCTCACA
CGACTGCTCGACCAATGGGCTGATCAGCTTCATCAAGAAGAACAGT

>node#4
CTCACCAGCGACCCCAACTTCCAGAAGCTGGAGCAATGGTACAAAGCCAACGCCGGCAGCCTCA
ACATGAGGCAGATGTTTGAGGCCGATAAGGAGAGGTTCAGCAAGTTCAGTACAACCCTTAAAACC
GATGATGGAGACATCCTGATCGATTATTCAAAGAACCTCATCACTGAGGAAGTCATGAAGATGCT
GTTCGATCTGGCAAAGTCGAGGGGAGTGGAGGAAGCCAGGGAGAGGATGTTCACTGGAGAGAA
GATCAACTTCACTGAGGGCCGAGCTGTGCTCCACATTGCCCTGAGGAACCGCTCCAACACTCCC
ATCCACGTGGACGGCAAGGACGTCATGCCTGAGGTCAACAACGTCCTGGAGAAGATGAAGGGCT
TCTGTCAGAAAGTGCGCAGTGGAGAGTGGAAGGGCTACACTGGAAAAGCCATCACTGATGTTGT
CAACATTGGCATTGGTGGATCTGACCTGGGCCCCTTGATGGTGACTGAAGCTCTGAAGCCCTACT
CCAAGGGAGGACCCCGGGTCTGGTTTGTCTCCAACATCGATGGCACTCACATGGCCAAGACCCT
GGCTGAGCTGAACCCTGAGACCACCCTCTTCATCATTGCATCCAAGACCTTCACCACCCAGGAGA
CCATCACCAATGCAGAGTCCGCCAAGGAGTGGTTCCTGCAGAAAGCTGGAGATAAATCTGCTGT
GGCCAAGCACTTTGTGGCTCTCTCCACCAATGCTCCCAAAGTGAAGGATTTCGGGATTGACACGA
ACAACATGTTTGAGTTCTGGGATTGGGTTGGTGGCCGTTATTCCCTGTGGTCAGCTATTGGCTTG
TCCATTGCTCTGCACATCGGCTTTGAGAACTTTGAGCAGCTGCTGAGTGGAGCTCACTGGATGGA
CAATCACTTCCGCACCGCCCCCCTGGAGAAGAACGTCCCCGTCCTGCTGGCCCTGCTGGGGGT
CTGGTACATCAACTTCTTCCAGGCGGAGACCCACGCCCTGCTGCCCTACGACCAGTACATGCAC
CGCTTTGCTGCCTACTTCCAGCAGGGGGACATGGAGTCCAATGGGAAGTACATCACCAGTAAAG
GCACCCGTGTGAACTACCACACTGGGCCCATCGTGTGGGGAGAGCCTGGGACCAATGGGCAGC
ATGCCTTCTACCAGCTCATCCATCAAGGAACACGCATGATTCCTGCTGACTTCCTCATCCCTGTC
CAGACTCAGCACCCCATCAGGAACAACCTGCACCACAAGATCCTGCTGGCTAACTTCCTGGCCC
AGACTGAGGCCCTGATGAAGGGGAAGACCACAGAGGAGGCCCGGAAGGAGCTGGAGGGGGCA
GGCCTGAGTGGAGAGGCCCTGGAGAAACTGCTGCCTCACAAGGTCTTCGAAGGAAACAAGCCAA

CCAACTCCATTGTCTTTAAGAAGCTGACTCCCTTCATGCTTGGAGCACTGATTGCCATGTATGAAC
ACAAGATCTTCGTGCAGGGTGTCATCTGGGACATCAACAGCTATGACCAGTGGGGAGTGGAACT
GGGGAAACAGCTGGCCAAGAAAATCGAGCCCGAGCTGCAGGATGCTAGCGAGGTGACCTCACA
CGACTGCTCGACCAATGGGCTGATCAGCTTCATCAAGAAGAACAGT

>node#5
CTCACAAGCGACCCCAACTTCCAGAAGCTGGAGCAATGGTACAAAGCCAACGCCGGAAACCTCA
ACATGAGGCAGATGTTTGAGGCCGACAAGGACAGATTCAGCAAGTTCAGTACAACACTGAAAACA
GATGATGGAGACATCCTGCTCGATTATTCCAAGAATCTCATCAATGAGGAAGTCATGAAGATGCT
GCTTGACCTGGCAAAGTCGAGGGGAGTGGAGGCTGCCAGGGAGAGGATGTTCACTGGAGAGAA
GATCAACTTCACTGAGGGCCGTGCTGTGCTCCACATTGCCCTGAGGAACCGCTCCAACACTCCC
ATCCATGTGGACGGGAAGGACGTCATGCCTGAGGTCAACAAGGTCCTGGAGAAGATGAAGGGCT
TCTGTCATAAAGTGCGCAGTGGAGAGTGGAAGGGCTACACTGGAAAAGCCATCACCGATGTTGT
CAATATCGGCATTGGTGGATCTGACCTGGGTCCCCTGATGGTGACTGAAGCTCTGAAGCCCTACT
CCAAGGGAGGACCCCGTGTCTGGTTTGTCTCCAACATCGATGGAACCCACATGGCCAAGACCCT
GGCTGAGCTGAACCCTGAGACCACCCTCTTCATCATTGCCTCCAAGACATTCACCACCCAGGAGA
CCATCACCAATGCAGAGTCTGCCAAGGAGTGGTTCCTGCAGACCGCTAAAGATAAATCTGCTGTG
GCCAAGCACTTTGTGGCTCTCTCCACCAATGCGCCCAAAGTGAAGGACTTCGGGATTGACACGG
ACAACATGTTTGAGTTCTGGGATTGGGTCGGTGGCCGTTATTCCCTGTGGTCAGCTATTGGCCTG
TCCATTGCTCTGCACATTGGCTTTGAGAACTTTGAGCAGCTGCTGAGTGGAGCTCACTGGATGGA
CAACCACTTCCGCACCGCTCCCCTGGAGAAGAACGTCCCCGTCCTGCTGGCCCTGCTGGGGGT
CTGGTACATCAACTTCTTCCAGGCGGAGACCCACGCCCTGCTGCCCTACGACCAGTACATGCAC
CGCTTCGCTGCCTACTTCCAGCAGGGGGACATGGAGTCCAATGGGAAGTACATCACCAACCAGG
GCACCCGTGTGAACTACCACACTGGGCCCATCGTGTGGGGAGAGCCAGGGACCAACGGACAGC
ATGCCTTCTACCAGCTCATCCACCAAGGAACACGCATGATTCCTGCTGACTTCCTCATCCCTGCC
CAGACCCAGCACCCCATCAGAAACAACCTGCACCACAAGATCCTGATGGCCAACTTCCTGGCCC
AGACTGAGGCCCTGATGAAGGGGAAGACCACAGAGGAGGCCAGGAAGGAGCTGGAGGCCGGC
GGCCTGAGCGGAGAGGCCCTGGAGAAGCTGCTGCCGCACAAAGTATTCGAAGGAAACAAGCCA
ACCAACTCCATTGTCTTCAAGAAGCTGACCCCCTTCATCCTCGGAGCACTTATTGCCATGTATGA
GCACAAGATCTTCGTGCAGGGTGTCATGTGGGAGATCAACAGTTATGACCAGTGGGGAGTGGAG
CTGGGGAAACAGCTGGCCAAGAAGATTGAGCCCGAGCTGCAGGACGCTGCTGAGGTCAACTCC
CACGACTCCTCCACCAACGGGCTCATCAACTTCATCAAGAAGAACTTC

>node#6
CTCACAAGCGACCCGAACTTCCAGAAGCTGGAGCAATGGTACAAAGCCCACGCCGGGAACCTCA
ACATGAGGCAGATGTTTGAGGCCGACAAGGACAGATTCAGCAAGTTCAGTACAACACTGAAAACA
GATGATGGAGACATCCTGCTTGATTATTCCAAGAATCTCATCAATGAGGAAGTCATGAAGATGCT
GGTTGACCTGGCAAAGTCGAGGGGAGTGGAGGCTGCCAGGGAGAGGATGTTCACTGGAGAGAA
GATCAACTTCACTGAGGGCCGTGCTGTGCTCCACGTTGCCCTGAGGAACCGCTCCAACACTCCC
ATCATTGTGGACGGGAAGGACGTCATGCCTGAGGTCAACAAGGTCCTGGAGAAGATGAAGGGCT
TCTGTCATAAAGTGCGCAGTGGAGAGTGGAAGGGCTACACTGGAAAGGCCATCACAGATGTTGT
CAATATCGGCATTGGTGGATCTGACCTGGGTCCCCTGATGGTGACTGAAGCTCTGAAGCCCTACT
CCAAGGGTGGACCCCGTGTCTGGTTTGTCTCCAACATCGATGGAACGCACATCGCCAAGACCCT
GGCTGAGCTGAACGCTGAGACCACCCTCTTCATCATTGCCTCCAAGACATTCACCACCCAGGAG
ACCATCACCAATGCAGAGTCTGCCAAGGAGTGGTTCCTGCAGGCCGCTAAAGATAAATCTGCTGT
GGCCAAGCACTTTGTGGCTCTCTCCACCAATGCGCCCAAAGTGAAGGACTTCGGGATCGACACG
GAGAACATGTTTGAGTTCTGGGACTGGGTCGGTGGACGTTATTCCTTGTGGTCAGCTATTGGGCT
GTCCATTGCTCTGCACATTGGCTTTGAGAACTTTGAGAAGCTGCTGAGTGGAGCTCACTGGATGG
ACAACCACTTCCGCACCGCTCCCCTGGAGAAGAACGCCCCCGTCCTGCTGGCCCTGCTGGGGA
TCTGGTACATCAACTTCTTCCAGGCGGAGACCCACGCCCTGCTGCCCTACGACCAGTACATGCA
CCGCTTCGCTGCCTACTTCCAGCAGGGGGACATGGAGTCCAATGGGAAGTACATCACCAATCAG
GGCACCCGTGTGAACTACCACACCGGGCCCATCGTGTGGGGAGAGCCAGGAACCAACGGACAG
CATGCCTTCTACCAGCTCATCCACCAAGGAACACGCATGGTTCCTGCTGACTTCCTCATCCCTGC
CCAGACACAGCATCCCATCAGAAACAACCTGCACCACAAGATCCTGATGGCCAACTTCCTGGCC
CAGACTGAGGCCCTGATGAAGGGGAAGACCACAGAGGAGGCGAGGAAGGAGCTGGAGGCCGG
CGGCCTGAGCGGAGAGGCTCTGGAGAAACTGCTGCCGCACAAAGTATTCGAGGGAAACAAGCC
AACCAACTCCATTGTCTTCAAGAAGCTGACCCCGTTCATCCTCGGAGCACTTATTGCCATGTATGA
GCACAAGATCTTCGTGCAGGGTGTCATGTGGGAGATCAACAGTTTTGACCAGTGGGGAGTGGAG

CTGGGGAAACAGCTGGCCAAGAAGATTGAGCCCGAGCTGCAGGACGCTGCTGAGGTCAACTCC
CACGACTCCTCCACCAACGGGCTCATCAACTTCATCAAGAAGAACTTC

>node#7
CTCACAAGCGACCCCAACTTCCAGAAGCTGGAGCAATGGTACAAAGCCCACGCCGGGAACCTCA
ACATGAGGCAGATGTTTGAGGCCGACAAGGACAGATTCAGCAAGTTCAGTACAACACTGAAAACA
GATGATGGAGACATCCTGCTTGATTATTCCAAGAATCTCATCAATGAGGAAGTCATGAAGATGCT
GGTTGACCTGGCAAAGTCGAGGGGAGTGGAGGCTGCCAGGGAGAGGATGTTCACTGGAGAGAA
GATCAACTTCACTGAGGGCCGTGCTGTGCTCCACGTGGCCCTGAGGAACCGCTCCAACACTCCC
ATCATTGTGGACGGGAAGGACGTCATGCCTGAGGTCAACAAGGTCCTGGAGAAGATGAAGGGCT
TCTGTCATAAAGTGCGCAGTGGCGAGTGGAAGGGCTACACTGGAAAGGCCATCACAGATGTTGT
CAATGTCGGCATTGGTGGATCTGACCTGGGTCCCCTGATGGTGACTGAAGCTCTGAAGCCCTAC
TCCAAGGGTGGACCCCGTGTCTGGTTTGTGTCCAACATCGATGGAACGCACATCGCCAAGACCC
TGGCTGAGCTGAACGCTGAGACCACCCTCTTCATCATTGCTTCCAAGACATTCACCACCCAGGAG
ACCATCACCAATGCAGAGTCTGCCAAGGAGTGGTTCCTGCAGGCCGCCAAAGATAAATCTGCTG
TGGCCAAGCACTTTGTGGCTCTCTCCACCAATGGACCCAAAGTGAAGGACTTCGGGATCGACAC
GGAGAACATGTTTGAGTTCTGGGACTGGGTCGGTGGACGTTATTCCTTGTGGTCAGCCATTGGG
CTGTCCATTGCTCTGCACATTGGCTTTGAGAACTTTGAGAAGCTGCTGAGTGGAGCTCACTGGAT
GGACAACCACTTCCGCACCGCTCCCCTGGAGAAGAACGCCCCCGTCCTGCTGGCCCTGCTGGG
GATCTGGTACATCAACTTCTTCCAGGCGGAGACCCACGCCATGCTGCCCTACGACCAGTACATG
CACCGCTTCGCTGCCTACTTCCAGCAGGGGGACATGGAGTCCAATGGAAAGTACATCACCAATC
AGGGCACCCGCGTGAACTACCACACCGGGCCCATCGTGTGGGGAGAGCCAGGAACCAACGGAC
AGCATGCCTTCTACCAGCTCATCCACCAAGGAACACGCATGGTTCCTGCTGACTTCCTCATCCCT
GCCCAGACACAGCATCCCATCAGAAACAACCTGCACCACAAGATCCTGATGGCCAACTTCCTGG
CCCAGACTGAGGCTCTGATGAAGGGGAAGACCACAGAGGAGGCGAGGAAGGAGCTGGAGGCC
GGCGGCCTGAGCGGAGAGGCTCTGGAGAAACTGCTGCCGCACAAAGTATTCCAGGGAAACAAG
CCAACCAACTCCATTGTCTTCAAGAAGCTGACCCCGTTCATCCTCGGAGCACTTATTGCCATGTAT
GAGCACAAGATCTTCGTACAGGGTGTCATGTGGGAGATCAACAGTTTTGACCAGTGGGGAGTGG
AGCTGGGGAAACAGCTGGCCAAGAAGATTGAGCCCGAGCTGCAGGACGCTGCTGAGGTCAACT
CCCACGACTCCTCCACCAACGGGCTCATCAACTTCATCAAGAAGAACTTC

>node#8
CTCACAAGCGACCCCAACTTCCAGAAGCTGGAGAAATGGTACAAAGCCCACGCCGTGAACCTCA
ACATGAGGCAGATGTTTGAGGCCGACAAGGACAGATTCAACAAGTTCAGTACAACACTGAAAACA
GATGATGGAGACATTCTGCTGGATTATTCCAAGAATCTCATCAATGAGGAAGTCATGAAGATGCT
GGTTGACCTGGCAAAGTCGAGAGGCGTGGAGGCTGCCAGGGAGAGGATGTTCTCTGGAGAGAA
GATCAACTTCACTGAGGGCCGTGCTGTGCTCCATGTGGCCCTGAGGAACCGCTCCAACACTCCC
ATCATTGTGGACGGGAAGGACGTCATGCCGGAGGTCAACAAGGTTCTGGAGAAGATGAAGGGCT
TCTGTCATAAAGTCCGCAGTGGCGAGTGGAAGGGCTACACTGGAAAGGCCATCACAGATGTTGT
CAATGTCGGCATTGGTGGATCTGACCTGGGTCCCCTGATGGTGACTGAGGCTCTGAAGCCCTAC
TCCAAGGGTGGACCCCGTGTCTGGTTTGTGTCCAACATCGATGGAACGCACATCGCCAAGACCC
TGGCTGAGCTGAACGCTGAGACCACCCTCTTCATCATTGCTTCCAAGACATTCACCACCCAGGAG
ACCATCACCAACGCTGAGTCTGCCAAGGAGTGGTTCCTGCAGGCCGCCAAAGATAAATCTGCTG
TGGCCAAGCACTTTGTGGCTCTCTCCACCAATGGACCCAAAGTGAAGGACTTCGGCATCGACAC
GGAGAACATGTTTGAGTTCTGGGACTGGGTCGGTGGACGTTACTCCTTGTGGTCAGCCATTGGG
ATGTCCATTGCCCTGCACATTGGCTTTGAGAACTTTGAGAAGCTGCTGACTGGAGCTCACTGGAT
GGACAACCACTTCCGCACCGCTCCCCTGGAGAAGAACGCCCCCGTCCTGCTGGCCCTGCTGGG
GATCTGGTACATCAACTTCTTCCAGGCGGAGACCCACGCCATGCTGCCCTACGACCAGTACATG
CACCGCTTCGCTGCCTACTTCCAGCAGGGGGACATGGAGTCCAATGGAAAGTACATCACCAATC
AGGGCACACGTGTGAACTACCACACCGGGCCCATCGTGTGGGGAGAGCCAGGAACCAACGGAC
AGCATGCCTTCTACCAGCTCATCCACCAAGGAACACGCATGGTTCCTGCTGACTTCCTCATCCCT
GCCCAGACACAGCATCCCATCAGAAACAACCTGCACCACAAGATCCTGATGGCCAACTTCCTGG
CCCAGACTGAGGCTCTGATGAAGGGGAAGACCACAGAGGAGGCCAGGAAGGAGCTGGAGGCTG
GCGGCCTGAGCGGAGAGGCTCTGGAGAAACTGCTGCCACACAAAGTATTCCAGGGAAACAAGC
CAACCAACTCAATTGTCTTCAAGAAGCTGACCCCGTTCACCCTCGGAGCACTTATTGCCATGTAT
GAGCACAAGATCTTCGTCCAGGGTGTCATGTGGGAGATCAACAGTTTTGACCAGTGGGGAGTGG
AGCTGGGGAAACAGCTGGCGAAGAAGATTGAGCCTGAGCTGCAGGACGCCGCTGAGGTCAACT
CCCACGACTCCTCCACCAACGGGCTCATCAACTTCCTCAAGAAGAACTTC

>node#9
CTCACAAGCGACCCCAACTTCCAGAAGCTGGAGAAATGGTACAAAGCCCACGCCTTGAACCTCA
ACCTGAGGCAGATGTTTGAGGCTGACAAGGAGAGATTCAACAAGCTCAGTACAACACTGAAAACC
GATGATGGAGACATTCTTCTGGATTATTCCAAGAATCTCATCACTGAGGAAGTCATGAAGATGCTG
GTTGACCTGGCAAAGTCGAGAGGCGTGGAGGCTGCCAGGGAGAGGATGTTCTCTGGAGAGAAG
ATCAACTTCACTGAGGGCCGTGCTGTGCTCCATGTGGCTCTGAGGAACCGCTCCAACACTCCCA
TCATTGTTGACGGGAAGGATGTCATGCCAGAAGTCAACAAGGTTCTGGAGAAGATGAAGGGCTT
CTGTCATAAAGTTCGCAGTGGCGAGTGGAAGGGCTACACTGGAAAGGCCATCACAGATGTTGTC
AATGTTGGCATTGGTGGATCTGACCTGGGTCCCCTGATGGTGACTGAGGCTCTGAAGCCCTACT
CCAAGGGTGGACCCCGCGTGTGGTTTGTGTCCAACATCGATGGAACCCACATTGCCAAGACCCT
GGCTCAGCTGAATGCTGAGACCACCCTCTTCATCATCGCTTCCAAGACATTCACCACCCAAGAGA
CCATCACCAACGCTGAGTCTGCCAAGGAATGGTTCCTCCAGGCCGCCAAAGATAAATCTGCTGT
GGCCAAGCACTTTGTGGCTCTCTCCACCAATGGCCCCAAAGTGAAGGACTTCGGCATCGACACA
GAGAACATGTTTGAGTTCTGGGACTGGGTCGGTGGACGTTTCTCCTTGTGGTCAGCCATTGGGAT
GGCCATTGCCTTGCACATTGGCTTTGACAACTTTGAGAAGCTTCTGTCTGGAGCTCACTGGATGG
ACAACCACTTCCGCACCGCTCCCCTGGACAAGAACGCCCCCGTCCTGCTGGCTCTGCTGGGCAT
CTGGTACATCAACTTCTTCCATGCTGAGACCCACGCCATGCTGCCCTACGACCAGTACATGCACC
GCTTCGCTGCCTACTTCCAGCAGGGGGACATGGAGTCCAATGGAAAGTACATCACAAACCATGG
CGCACGTGTGAACTACCACACCGGGCCCATCGTGTGGGGAGAGCCAGGAACCAACGGGCAGCA
CGCCTTCTACCAGCTCATCCACCAAGGAACACGCATGGTTCCTGCTGACTTCCTCATCCCTGCCC
AGACACAGCATCCCATCAGAGACAACCTGCACCACAAGATCTTGCTGGCCAACTTCCTGGCCCA
GACTGAGGCTCTGATGAAGGGGAAGACCACAGAGGAGGCCAGGAAGGAGCTGGAGGCTGGCG
GCCTGAGCGGAGAGGCTCTGGAGAAACTGCTGCCACACAAAGTATTCCAGGGAAACAAGCCAAC
CAACTCAATCATCTTCAAGAAGCTGACTCCGTTCACACTCGGAGCACTTATTGCCATGTATGAGCA
CAAGATCTTCGTCCAGGGTGTCATGTGGGAGATCAACAGTTTTGACCAGTGGGGAGTCGAGCTG
GGCAAACAGCTGGCGAAGAAGATTGAGCCTGAGCTGCAGGACGCGGCTGAGGTCAACTCCCAC
GACTCCTCCACCAACGGGCTCATCAACTTCCTCAAGAAGAACTTC

>node#10
CTCACACACGACCCCAACTTCCAAAAGCTGCAGAACTGGTACACAGCCCACGCCTTGAACCTCAA
CCTGAGGCACATGTTTGAGGCTGACAAGGAGAGATTCAACAAGCTCAGTCTCACACTGAAAACTG
ATGATGGAGACATTCTTCTGGATTATTCCAAGAATCTCATCACTGACGAAGTCGTGAAGATGCTG
GTTGACCTGGCCAAGTCCAGAGGCATTGAGGCTGCCAGGGAGAGGATGTTCTCAGGAGAGAAG
ATCAACTTCACTGAGGGCCGTGCTGTGCTCCATGTGGCCCTGAGGAACCGCTCCAACACTCCCA
TCATTGTTGACGGCAAGGATGTGATGCCAGAAGTCAACAAAGTCCTGGAGAAGATGAAGGGCTT
CTGTCATAAAGTTCGCAGTGGCGAGTGGAAGGGCTACACGGGAAAGGCCATCACAGATGTTGTC
AATGTCGGCATTGGTGGATCTGACCTTGGTCCCCTGATGGTGACTGAGGCCCTGAAGCCGTACT
CCAAGGGTGGACCCCGCGTGTGGTTTGTGTCCAACATTGATGGAACCCACATTGCCAAGACCCT
TGCACAGCTGAATGCTGAGACCACCCTCTTCATCATCGCTTCCAAGACATTCACCACCCAAGAGA
CCATCACCAACGCTGAGTCGGCCAAAGCATGGTTCCTCGAGCACGCCAAAGATAAAGCTGCTGT
GGCCAAGCACTTTGTGGCTCTTTCCACCAATGGCCCCAAAGTGAAGGACTTCGGCATTGACACA
GAGAACATGTTTGAGTTCTGGGATTGGGTCGGTGGCCGTTTCTCCTTGTGGTCTGCCATTGGAAT
GGCCATTGCTTTGCACATTGGCTTTGACAACTTTGAGAAGCTTCTGTCAGGAGCTCACTGGATGG
ACAACCACTTCCGCACCGCTCCCCTGGATAAGAACGCTCCCGTCCTGCTGGCTCTGCTGGGCAT
CTGGTACATCAACTTCTTCCATGCTGAGACCCACGCCATGCTGCCTTACGATCAGTACATGCACC
GCTTCGCCGCCTACTTCCAGCAGGGGGACATGGAGTCAAATGGAAAGTACATCACAAACCATGG
AGCACGTGTGAACTACCACACCGGGCCGATCGTGTGGGGAGAGCCAGGAACCAACGGGCAGCA
CGCCTTCTACCAGCTCATCCACCAAGGAACACGCATGGTGCCTGCTGACTTCCTGATCCCAGCTC
AGACACAGCATCCCATCAGAGACAACCTGCACCACAAGATCTTGTTGGCCAACTTCCTGGCCCAG
ACCGAGGCCCTGATGAAGGGTAAGACCACAGAGGAGGCCAGGAAGGAGCTGGAGGCGGGCGG
CCTGAGCGGAGAGGCTCTGGAGAAAATTCTGCCACACAAAGTATTCCAGGGAAACAAGCCAACC
AACTCAATCATCTTCAAGAAGCTGACCCCGTTCACACTCGGAGCACTTATAGCAATGTATGAGCA
CAAGATCTTCGTCCAGGGTGTAATGTGGGAGATCAACAGTTTTGACCAGTGGGGAGTCGAACTG
GGCAAACAGCTGGCGAAGAAGATTGAGCCTGAGCTCAAGGACGCGGCGGAGGTCAACTCCCAC
GACTCCTCCACCAACGGGCTCATCAACTTCCTCAAGAAGAACTTT

>node#11
CTCACAAGCGACCCCAACTTCCAGAAGCTGGAGCAATGGTACAAAGCCAACGCCGGAAACCTCA

ACATGAGGCAGATGTTTGAGGCCGACAAGGACAGATTCAGCAAGTTCAGTACAACACTGAAAACA
GATGATGGAGACATCCTGCTCGATTATTCCAAGAATCTCATCAATGAGGAAGTCATGAAGATGCT
GCTTGACCTGGCAAAGTCGAGGGGAGTGGAGGCTGCCAGGGAGAGGATGTTCACTGGAGAGAA
GATCAACTTCACTGAGGGCCGTGCTGTGCTCCACATTGCCCTGAGGAACCGCTCCAACACCCCC
ATCCATGTGGACGGGAAAGACGTCATGCCTGAGGTCAACAGGGTCCTGGAGAAGATGAAGGGCT
TCTGTCATAAAGTGCGCAGTGGAGAGTGGAAGGGCTACACTGGAAAAGCCATCACCGATGTTGT
CAATATCGGCATTGGTGGATCTGACCTGGGTCCCCTGATGGTGACTGAAGCTCTGAAGCCCTACT
CCAAGGGAGGACCCCGTGTCTGGTTTGTCTCCAACATCGATGGAACCCACATGGCCAAGACCCT
GGCTGAGCTGAACCCTGAGACCACCCTCTTCATCATTGCCTCCAAGACATTCACCACCCAGGAGA
CCATCACCAATGCAGAGTCTGCCAAGGAGTGGTTCCTGCAGACCGCTAAAGATAAATCTGCTGTG
GCCAAGCACTTTGTGGCTCTCTCCACCAATGCGCCCAAAGTGAAGGACTTTGGGATTGACACGG
ACAACATGTTTGAGTTCTGGGATTGGGTCGGTGGCCGTTATTCCCTGTGGTCAGCTATTGGCCTG
TCCATCGCTCTGCACATTGGCTTTGAGAACTTTGAGCAGCTGCTGAGTGGAGCTCACTGGATGGA
CAACCACTTCCGCAGCGCTCCCCTGGAGAAGAACGTCCCCGTCCTGCTGGCCCTGCTGGGGGT
CTGGTACATCAACTTCTTCCAGGCGGAGACCCACGCCCTGCTGCCCTACGACCAGTACATGCAC
CGCTTCGCTGCCTACTTCCAGCAGGGGGACATGGAGTCCAATGGGAAGTACATCACCAACCAGG
GCACCCGTGTGAACTACCACACTGGGCCCATCGTGTGGGGAGAGCCAGGGACCAACGGACAGC
ATGCCTTCTACCAGCTCATCCACCAAGGAACACGCATGATTCCTGCTGACTTCCTCATCCCTGCC
CAGACCCAGCACCCCATCAGAAACAACCTGCACCACAAGATCCTGATGGCCAACTTCCTGGCCC
AGACTGAGGCCCTGATGAAGGGGAAGACCACAGAGGAGGCCAGGAAGGAGCTGGAGGCCGGC
GGCCTGAGCGGAGAGGCCCTGGAGAAGCTGCTGCCGCACAAAGTATTCGAAGGAAACAAGCCA
ACCAACTCCATTGTCTTCAAGAAGCTGACCCCCTTCATCCTCGGAGCACTTGTTGCCATGTATGA
GCACAAGATCTTCGTGCAGGGTGTCATGTGGGAGATCAACAGTTATGACCAGTGGGGAGTGGAG
CTGGGGAAGCAGCTGGCCAAGAAGATTGAGCCCGAGCTGCAGGACGCTGCTGAGGTCAACTCC
CACGACTCCTCCACCAACGGGCTCATCAACTTCATCAAGAAGAACTTC

>node#12
CTCACAAGCGACCCGAACTTCCAGAAGCTGGAGCAATGGTACAAAGCCAACGCCGGAAACCTCA
ACATGAGGCAGATGTTTGAGGCCGACAAGGACAGATTCAGCAAGTTCAGTACAACACTGAAAACA
GATGATGGAGACATCCTGCTCGATTACTCCAAGAATCTCATCAATGAGGAAGTCATGAAGATGCT
GCTTGACATGGCAAAGTCGAGGGGAGTGGAGGCTGCCCGGGAGAGGATGTTCACTGGAGAGAA
GATCAACTTCACTGAGGGCCGTGCTGTGCTCCACATTGCCCTGAGGAACCGCTCCAACACCCCC
ATCCATGTGGACGGGAAAGACGTCATGCCTGAGGTCAACAGGGTCCTGGAGAAGATGAAGGGCT
TCTGTCATAAAGTGCGCAGTGGAGAGTGGAAGGGCTTCACTGGAAAAGCCATCACGGATGTTGT
CAATATCGGCATTGGTGGATCTGACCTGGGTCCTCTGATGGTGACTGAAGCTCTGAAGCCCTACT
CCAAGGGAGGACCCCGTGTCTGGTTTGTCTCCAACATCGATGGAACCCACATGGCCAAGACCCT
GGCTGAGCTGAACCCTGAGACCACCCTCTTCATCATTGCCTCCAAGACATTCACCACCCAGGAGA
CCATCACCAATGCAGAGTCTGCCAAGGAGTGGTTCCTGCAGACCGCTAAAGATAAATCTGCTGTG
GCCAAGCACTTTGTGGCTCTCTCCACCAATGCGCCCAAAGTGAAGGACTTTGGGATTGACACGG
ACAACATGTTTGAGTTCTGGGATTGGGTCGGAGGCCGTTATTCCCTGTGGTCAGCTATTGGCCTG
TCCATCGCTCTGCACATTGGCTTTGAGAACTTTGAGCAGCTTCTGAGTGGAGCTCACTGGATGGA
CAACCACTTCCGCAGCGCTCCCCTGGAGAAGAACGTCCCCGTCCTCCTGGCCCTGCTGGGGGT
CTGGTACATCAACTTCTTCCAGGCGGAGACCCACGCCCTGCTGCCCTACGACCAGTACATGCAC
CGCTTCGCTGCCTACTTCCAGCAGGGGGACATGGAGTCCAATGGGAAGTACATCACCAACCAGG
GCACCCGTGTGAACTACCACACCGGGCCCATCGTGTGGGGAGAGCCAGGGACCAACGGACAGC
ATGCCTTCTACCAGCTCATCCACCAAGGAACACGCATGATTCCTGCTGACTTCCTCATCCCTGCC
CAGACCCAGCACCCCATCAGAAACAACCTGCACCACAAGATCCTGATGGCCAACTTCCTGGCCC
AGACTGAGGCCCTGATGAAGGGGAAGACCACAGAGGAGGCCAGGAAGGAGCTGGAGGCCGGC
GGCCTGAGCGGAGAGGCCCTGGAGAAGCTGCTGCCGCACAAAGTATTCGAAGGAAACAAGCCA
ACCAACTCCATTGTCTTCAAGAAGCTGACCCCCTTCATCCTTGGAGCACTTGTTGCCATGTATGA
GCACAAGATCTTCGTGCAGGGTGTCATGTGGGAGATCAACAGTTATGACCAGTGGGGAGTGGAG
CTGGGGAAGCAGCTGGCCAAGAAGATTGAGCCCGAGCTGCAGGACGATGCTGAGGTCAACTCC
CACGACTCCTCCACCAACGGGCTCATCAACTTCATCAAGAAGAACTTC

>node#13
CTCACAAACGACCCCAACTTCCAGAAGCTGGAGCAATGGTACAAAGCCAACGCCGGAAACCTCA
ACATGAGGCAGATGTTTGAGGCCGACGAGGACAGATTCAGCAAGTTCAGTACGACCCTGCAGAC
AGATGATGGAGACATCCTGCTGGATTACTCCAAGAATCTCATCAATGAGGAAGTCATGAAGATGC

TGCTCGCCATGGCAAAGTCGAGGGGAGTGGAGGCGGCCCGGGAGAGGATGTTCTCTGGAGAGA
AGATCAACTTCACTGAGGGCCGTGCTGTGCTCCACATTGCCCTGAGGAACCGCTCCAACACCCC
CATCCTCGTGGACGGGAAAGACGTGATGCCTGAGGTCAACAGGGTCCTGGAGAAGATGAAGGG
CTTCTGTCATAAAGTGCGCAGTGGCGAGTGGAAAGGCTTCAGTGGGAAAGCCATCACCGATGTC
GTCAACATCGGCATTGGCGGCTCTGACCTGGGTCCTCTGATGGTGACTGAAGCTCTGAAGCCGT
ACTCCAAAGGAGGACCCAATGTCTGGTTCGTCTCCAACATCGATGGCACCCACATGGCCAAGAC
CCTGGCCAAGCTGAACGCTGAGACCACCCTCTTCATCATCGCCTCCAAGACATTCACCACCCAG
GAGACCATCACCAACGCAGAGTCTGCCAGGGAGTGGTTCCTGCAGACCGCTAAAGATAAATCTG
CTGTGGCCAAGCACTTTGTGGCTCTTTCCACCAATGCGCCCAAAGTGAAGGACTTTGGCATTGAC
ACGGACAACATGTTTGAGTTCTGGGATTGGGTCGGAGGTCGTTATTCCCTGTGGTCAGCTATTGG
TCTGTCCATCGCTCTGCACATAGGCTTTGAGAACTTTGAGCAGCTTCTGTCTGGAGCTCACTGGA
TGGACAACCACTTCCGCAGCGCTCCTCTGGAGAAGAACGTCCCCGTCCTCCTGGCCCTGCTGGG
CGTCTGGTACGTCAACTTCTTCCAGGCGGAGACCCACGCCCTGCTGCCCTACGACCAGTACATG
CACCGCTTCGCTGCGTACTTCCAGCAGGGGGACATGGAGTCCAACGGGAAGTACATCACCAAAG
ATGGCACCCGTGTGAACTACCACACCGGACCCATCGTGTGGGGAGAGCCAGGGACCAATGGAC
AGCATGCCTTCTACCAGCTCATCCACCAAGGAACTCGCATGATTCCTGCTGACTTCCTCATTCCT
GCCCAGACCCAGCATCCCATCAGAAACAACCTGCATCACAAGATCCTGATGGCCAACTTCCTGG
CCCAGACTGAGGCTCTGATGAAGGGGAAGACCACAGAGGAGGCCAGGAAGGAGCTGGAGGCC
GCCGGCCTGAGCGGAGACGCCCTGGAGAAGCTGCTGCCTCACAAAGTATTCGAAGGAAACAAG
CCAAGCAACTCCATTGTCTTCAAGAAGCTGACTCCCTTCATCCTGGGAGCCCTGGTTGCCATGTA
TGAGCACAAGATCTTCGTGCAGGGCGTCATGTGGGACATCAACAGTTATGACCAGTGGGGAGTC
GAGCTGGGGAAGCAACTGGCCAAGAAGATTGAGCCCGAGCTGCAGGACGATTCTGAGGTCAACT
CCCACGACTCCTCCACCAACGGGCTCATCAACTTCCTCAAGAAGAACTTT

>node#14
CTCACAAACGACCCCAACTTCCAGAAGCTGGAGCAATGGTACAAAGCCAACGCCGGAAACCTCA
ACATGAGGCAGATGTTTGAGGCCGACGAGGACAGATTCAGCAAGTTCAGCACGACCCTGCAGAC
AGATGATGGAGACATCCTGCTGGATTACTCCAAGAATCTCATCAATGAGGAAGTCATGAAGATGC
TGCTCGCCATGGCAAAGTCGAGGGGGGTGGAGGAGGCCCGGGAGAGGATGTTCTCTGGAGAGA
AGATCAACTTCACTGAGGGCCGTGCTGTGCTCCACATTGCCCTGAGGAACCGCTCCAACACCCC
CATCCTCGTGGACGGGAAAGACGTGATGCCTGAGGTCAACAGGGTCCTGGAGAAGATGAAGGC
CTTCTGTCATAAAGTGCGCAGTGGCGAGTGGAAAGGCTTCAGTGGGAAAGCCATCACCGATGTC
GTCAACATCGGCATTGGCGGCTCTGACCTGGGTCCTCTGATGGTGACTGAAGCTCTGAAGCCGT
ACTCCAAAGGAGGACCCAATGTCTGGTTCGTCTCCAACATCGATGGCACCCACATGGCCAAGAC
CCTGGCCAAGCTGAACGCTGAGACCACCCTCTTCATCATCGCCTCCAAGACATTCACCACCCAG
GAGACCATCACCAACGCAGAGTCTGCCAGGGAGTGGTTCCTGCAGACCGCTAAAGATAAATCTG
CTGTGGCCAAGCACTTTGTGGCTCTTTCCACCAATGCGCCCAAAGTGAAGGACTTTGGCATTGAC
ACGGACAACATGTTTGAGTTCTGGGATTGGGTCGGAGGTCGTTATTCCCTGTGGTCAGCTATTGG
TCTGTCCATCGCTCTGCACATAGGCTTTGAGAACTTTGAGCAGCTTCTGTCTGGAGCTCACTGGA
TGGACAACCACTTCCGCAGCGCTCCTCTGGAGAAGAACGTCCCCGTCCTCCTGGCCCTGCTGGG
CGTCTGGTACGTCAACTTCTTCCAGGCGGAGACCCACGCCCTGCTGCCCTACGACCAGTACATG
CACCGCTTCGCTGCGTACTTCCAGCAGGGGGACATGGAGTCCAACGGGAAGTACATCACCAAAG
ATGGCACCCGTGTGAACTACCACACCGGACCAATCGTGTGGGGAGAGCCAGGGACCAATGGAC
AGCATGCCTTCTACCAGCTCATCCACCAAGGCACTCGCATGATTCCTGCTGACTTCCTCATTCCT
GCCCAGTCCCAGCATCCCATCAGAAACAACCTGCATCACAAGATCCTGATGGCCAACTTCCTGGC
CCAGACTGAGGCTCTGATGAAGGGGAAGACCACAGAGGAGGCCAGGAAGGAGCTGGAGGCCG
CCGGCCTGAAAGGAGACGCCCTGGAGAAGCTGCTGCCTCACAAAGTATTCGAAGGAAACAAGCC
AAGCAACTCCATCGTCTTCAAGAAGCTGACTCCCTTCATCCTGGGAGCCCTGGTTGCCATGTACG
AGCACAAGATCTTCGTGCAGGGCGTCATGTGGGACATCAACAGTTATGACCAGTGGGGAGTTGA
GCTGGGGAAGCAACTGGCCAAGAAGATTGAGCCCGAGCTGCAGGACGATTCTGAGGTCAACTCC
CACGACTCCTCCACCAACGGGCTCATCAACTTCCTGAAGAAGAACTTT

>node#15
CTCACCAACGACCCCAACTTCCAGAAGCTGCAGCAGTGGTACAAAGCCAACGCCGGAAACCTCA
ACATGAGGGAGATGTTCGAGGCCGACGAGGACAGATTCAGCAAGTTCAGCACGACTCTGCAGAC
GGATGATGGAGACATCCTCCTGGACTTCTCCAAGAATCTCATCAACCAGGAAGTCCTGGAGATGC
TGCTCGCTATGGCCAAGTCGAGGGGGGTGGAGGAGGCCCGGGAGAGGATGTTCTCTGGAGAGA
AGATCAACTTCACAGAGGGCCGTGCTGTGCTCCACGTTGCCCTGAGGAACCGCTCCAACACCCC

CATCCTCGTGGACGGGAAAGACGTGATGCCTGAGGTCAACCGGGTCCTGGAGAAGATGAAGGC
CTTCTGTCATAAAGTGCGCAGCGGCGAGTGGAAAGGCTTCAGTGGGAAAAGCATCACCGACGTG
GTCAACATCGGCATCGGCGGCTCTGACCTGGGTCCTCTGATGGTGACCGAGGCTCTGAAGCCGT
ACTCCACAGGAGGACCGAATGTCTGGTTCGTCTCCAACATCGATGGCACCCACATGGCCAAGAC
CCTGGCCAAGCTGGACGCTGAGACCACCCTGTTCATCATCGCCTCCAAGACATTCACCACCCAG
GAGACCATCACCAACGCGGAGTCGGCCAGAGACTGGTTCCTGCAGACCGCTAAAGATAAATCTG
CTGTGGCCAAGCACTTTGTGGCTCTTTCCACCAACGCGCCCAAAGTGAAGGACTTTGGCATTGAC
ACGGAGAACATGTTTGAGTTCTGGGATTGGGTCGGAGGTCGTTACTCCCTGTGGTCGGCCATCG
GTCTGTCCATCGCTCTGCACATAGGCTTTGAGAACTTTGAGCAGCTTCTGTCTGGAGCTCACTGG
ATGGACAACCACTTCCGCAGCGCTCCTCTGGAGAAGAACGTCCCCGTCCTCCTGGCCCTGCTGG
GCGTCTGGTACGTCAACTTCTTCCAGGCGGAGACCCACGCCCTGCTGCCCTACGACCAGTACAT
GCACCGCTTCGCCGCGTACTTCCAGCAGGGGGACATGGAGTCCAACGGGAAGTACATCACCAAA
GATGGCACCCGAGTGAACTACCACACCGGACCAATCGTGTGGGGAGAGCCAGGGACCAATGGA
CAGCATGCCTTCTACCAGCTCATCCACCAAGGAACTCGTATGATTCCTGCTGACTTCCTCATTCCT
GCTCAGTCTCAGCATCCAATCAGAAACAACCTGCATCACAAGATCCTGGTGGCCAACTTCCTGGC
TCAGACTGAAGCTCTGATGAAGGGGAAGACCACAGAGGAGGCGCGGAAGGAGCTGGAGGCCGC
CGGCCTGAAAGGAGACGCCCTGGAGAAGCTGCTGCCTCACAAAGTATTTGAAGGAAACAAGCCA
AGCAACTCCATCGTCTTCAAGAAGCTGACTCCGTTCATCCTGGGAGCTCTGGTTGCCATGTACGA
GCACAAGATCTTCGTGCAGGGCGTCATGTGGGACATCAACAGCTACGACCAGTGGGGCGTTGAG
CTGGGGAAGCAACTGGCCAAGAAGATTGAGCCGGAGCTGCAGGACGACTCTGAGGTCAACTCC
CACGACTCCTCCACCAACGGACTCATCAACTTCCTGAAGAAGAACTTT
